# Reconstitution of a Reversible Membrane Switch via Prenylation by One-Pot Cell-Free Expression

**DOI:** 10.1021/acssynbio.2c00406

**Published:** 2022-11-29

**Authors:** Lei Kai, Sonal, Tamara Heermann, Petra Schwille

**Affiliations:** † Department of Cellular and Molecular Biophysics, Max Planck Institute of Biochemistry, D-82152 Martinsried, Germany; ‡ School of Life Sciences, Jiangsu Normal University, Shanghai Road 101, 221116 Xuzhou, P. R. China; § Biosciences Division, University College London, Gower Street, WC1E 6BT London, U.K.

**Keywords:** synthetic biology, synthetic cell, reversible membrane switch, cell-free protein synthesis, prenylation, Cdc42

## Abstract

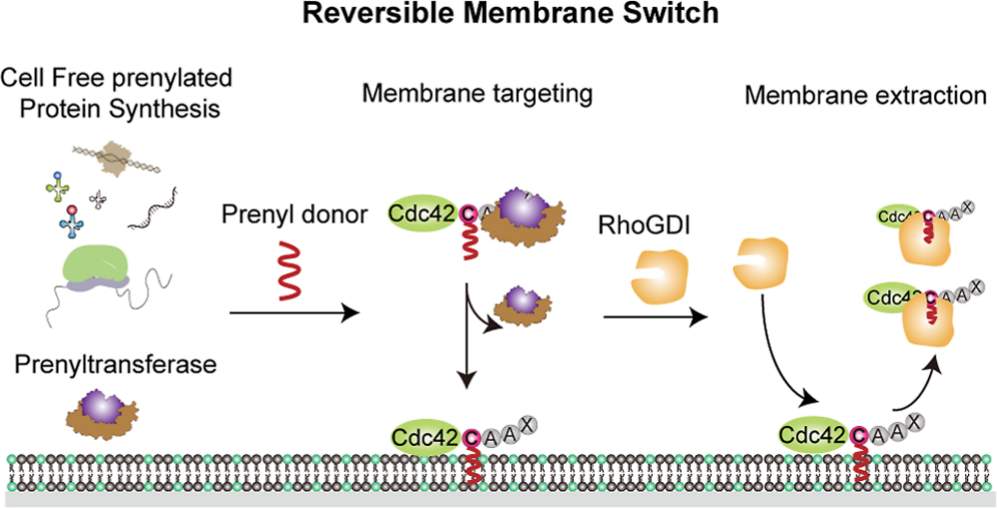

Reversible membrane targeting of proteins is one of the key regulators of cellular interaction networks, for example, for signaling and polarization. So-called “membrane switches” are thus highly attractive targets for the design of minimal cells but have so far been tricky to reconstitute in vitro. Here, we introduce cell-free prenylated protein synthesis (CFpPS), which enables the synthesis and membrane targeting of proteins in a single reaction mix including the prenylation machinery. CFpPS can confer membrane affinity to any protein via addition of a 4-peptide motif to its C-terminus and offers robust production of prenylated proteins not only in their soluble forms but also in the direct vicinity of biomimetic membranes. Thus, CFpPS enabled us to reconstitute the prenylated polarity hub Cdc42 and its regulatory protein in vitro, implementing a key membrane switch. We propose CFpPS to be a versatile and effective platform for engineering complex features, such as polarity induction, in synthetic cells.

## Introduction

In our pursuit to understand the fundamental principles of living systems, their compositional complexity and inherent redundancies are posing a notorious challenge. Hence, in recent years, attempts have been made to extrapolate the essential functional requirements for the emergence of biological systems by their bottom-up synthesis from (bio)chemical systems. This field, known as bottom-up synthetic biology, ultimately aims at the reconstitution of the simplest artificial system that mimics life.^1^ To this end, both biological and biology-inspired synthetic building blocks are enclosed in cell-sized compartments, consisting of self-assembled amphiphilic molecules, to create biomimetic or protocell-like systems.^2−5^ Biomembranes, considered to be essential elements of life, do not only act as physical boundaries in these compartments but also as matrices and catalytic interfaces for many cellular processes. In particular, dynamic interactions or reversible switch-like membrane targeting of signaling proteins allows to locally activate intracellular molecular pathways in response to diverse extracellular stimuli, which play a vital role in proliferation, differentiation, apoptosis, and division.^6^ Despite their important role, the reconstitution of such reversible membrane-targeting systems has proven extremely challenging, mainly due to the complexity of most of these multi-protein and multi-module systems,^7,8^ but also because of the notorious physicochemical challenges of maintaining functionality of large amphiphilic biopolymers in cell-free environments.

**Figure 1:**
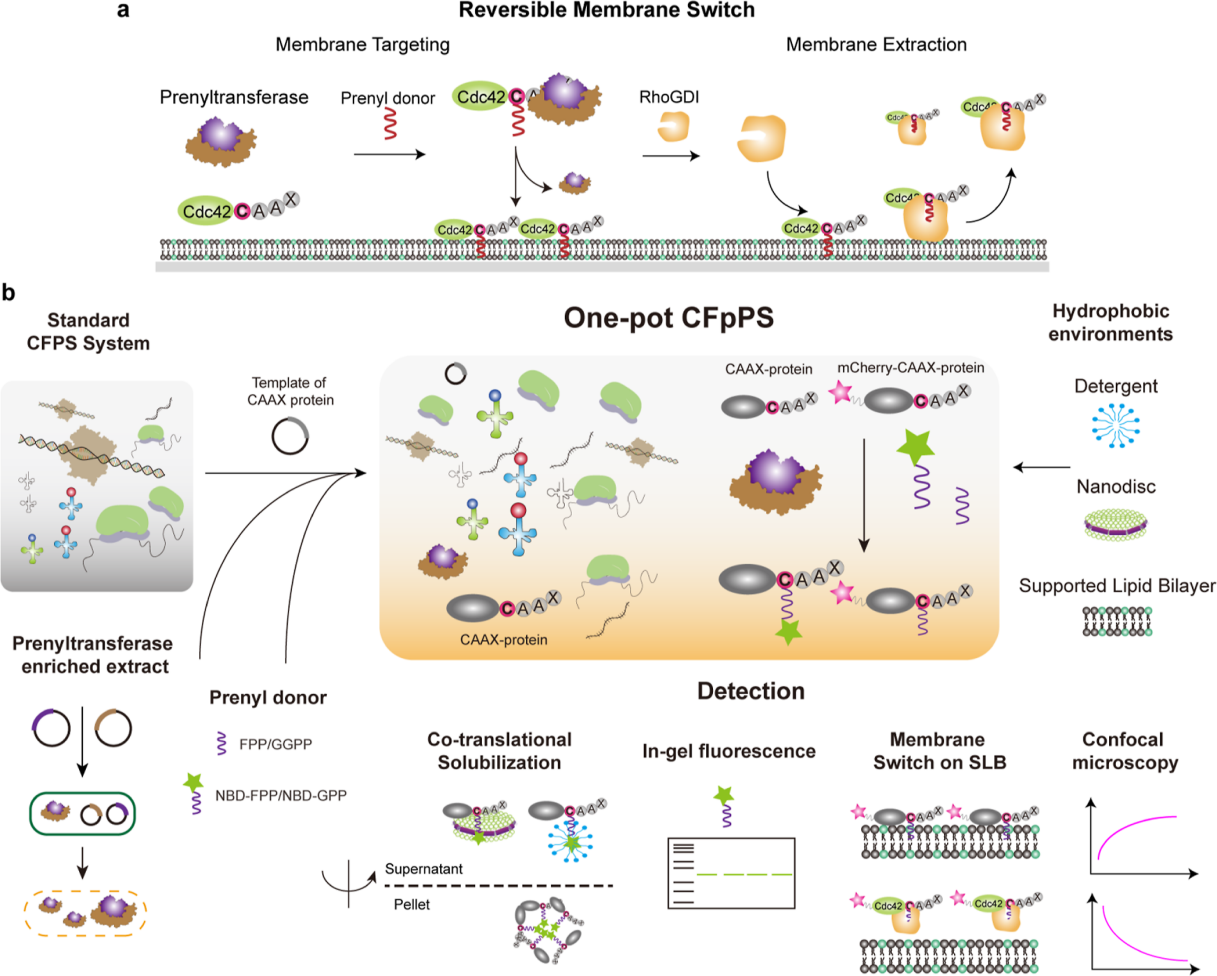
Schematic illustration of CFpPS and its application for the reconstitution of a reversible membrane switch. (a) Schematic of the reversible membrane switch process of Cdc42 via prenylation and regulator protein RhoGDI. (b) Co-translational prenylation was achieved by introducing prenyltransferase-enriched extract, the isoprenoid prenyl donor and the plasmid carrying the template of a target CAAX protein to the normal CFPS system. Newly expressed and prenylated proteins can either be directly incorporated into biomimetic membranes such as SLBs or solubilized with amphipathic reagents, such as detergents or nanodiscs. Prenylation efficiency can be monitored in real time through confocal microscopy with fluorescent fusion proteins or detected via in-gel fluorescence using fluorescent prenyl donors. Upon introduction of RhoGDI, a membrane switchable system could be established and monitored in real time.

Among the cellular membrane-switch systems that have been described in great detail are the *Escherichia coli* MinCDE system^9,10^ and the eukaryotic polarity hub protein Cdc42.^11−13^ In the former system, ATP-induced dimerization of ATPase MinD enhances its membrane affinity via an amphipathic membrane targeting sequence. Stimulation of ATP hydrolysis by its regulator protein MinE results in dissociation of membrane-attached MinD dimers and their subsequent detachment from the membrane.^14^ Following the same principle, a minimal synthetic phosphorylation-dependent membrane switch was recently reported, enabling the reversible membrane targeting via phosphorylation/dephosphorylation of designed heterodimeric coiled-coil peptides.^15^ In contrast, the Cdc42-based membrane switch system is biochemically more complex and, thus, harder to reconstitute. Here, membrane targeting is triggered by prenylation, a post-translation modification, which adds a prenyl group at a specific amino acid residue at the C-terminus of a protein.^16^ Membrane dissociation is promoted via specific guanine nucleotide Rho GDP dissociation inhibitors (RhoGDIs), which sequester the prenyl moiety, leading to the active extraction of prenylated Cdc42 from the membrane (Figure 1a). Although the mechanistic details of polarity establishment in eukaryotes are still being explored,^17,18^ it is well recognized that such switchable membrane-targeting of Cdc42, leading to its differential mobility on membranes versus in the cytoplasm, provides an important physical–chemical cue for downstream processes.^11^ However, the bottom-up assembly of this particular membrane switch has remained a great challenge due to several factors. First, overexpression and purification of homogenous prenylated Cdc42 have been extremely difficult due to the increased hydrophobicity from prenylation. Furthermore, the highly dynamic nature of functional Cdc42 in its purified form results in protein instability and aggregation in the absence of any regulatory proteins.^19,20^

One technology that has greatly facilitated the assembly of multi-module systems is cell-free protein synthesis (CFPS).^21,22^ CFPS systems host both the transcription and translation machineries, thus mimicking the cytoplasmic environment for protein production and facilitating a scale-up of functional modules in reconstituted systems. Recent advances in CFPS, in particular the well-established *E. coli*-based CFPS systems, have led to a highly efficient and versatile platform for bottom-up synthetic biology.^7^ We leveraged CFPS technology to overcome the challenges of reconstituting the Cdc42-based reversible membrane-switch system by developing the cell-free prenylated protein synthesis (CFpPS) system, wherein we integrated the eukaryotic prenylation machinery into the *E. coli*-based CFPS system (Figure 1b). This allows the synthesis and direct membrane targeting of Cdc42 in a single-pot reaction configuration. We have produced extracts enriched with prenylation enzymes, systematically investigated the functionality of these extracts, and successfully demonstrated the co-translational prenylation of a range of representative CAAX proteins—Kras, Hras, RhoA, RhoC, Rac1 and, in particular, Cdc42 from the Rho family—thus establishing a versatile system for cell-free synthesis of prenylated proteins. Furthermore, the co-translational solubilization of prenylated protein was achieved by introducing different solubilizing agents, such as detergents or lipid-based scaffolds, directly into the one-pot reaction. The reaction could also be carried out in the vicinity of a supported lipid bilayer (SLB) for direct assessment by confocal microscopy. Finally, CFpPS enabled reconstitution of a minimal reversible membrane-targeting system based on Cdc42 and RhoGDI—two components from eukaryotic polarity machinery. Furthermore, the Cdc42–RhoGDI module could act as a minimal carrier, dynamically shuttling a model protein (i.e., mCherry) between membrane and solution in a switch-like fashion. Thus, we demonstrate that the CFpPS system holds immense potential for bottom-up assembly of key biological processes such as cell polarization.

## Results and Discussion

### Establishing CFpPS

**Figure 2:**
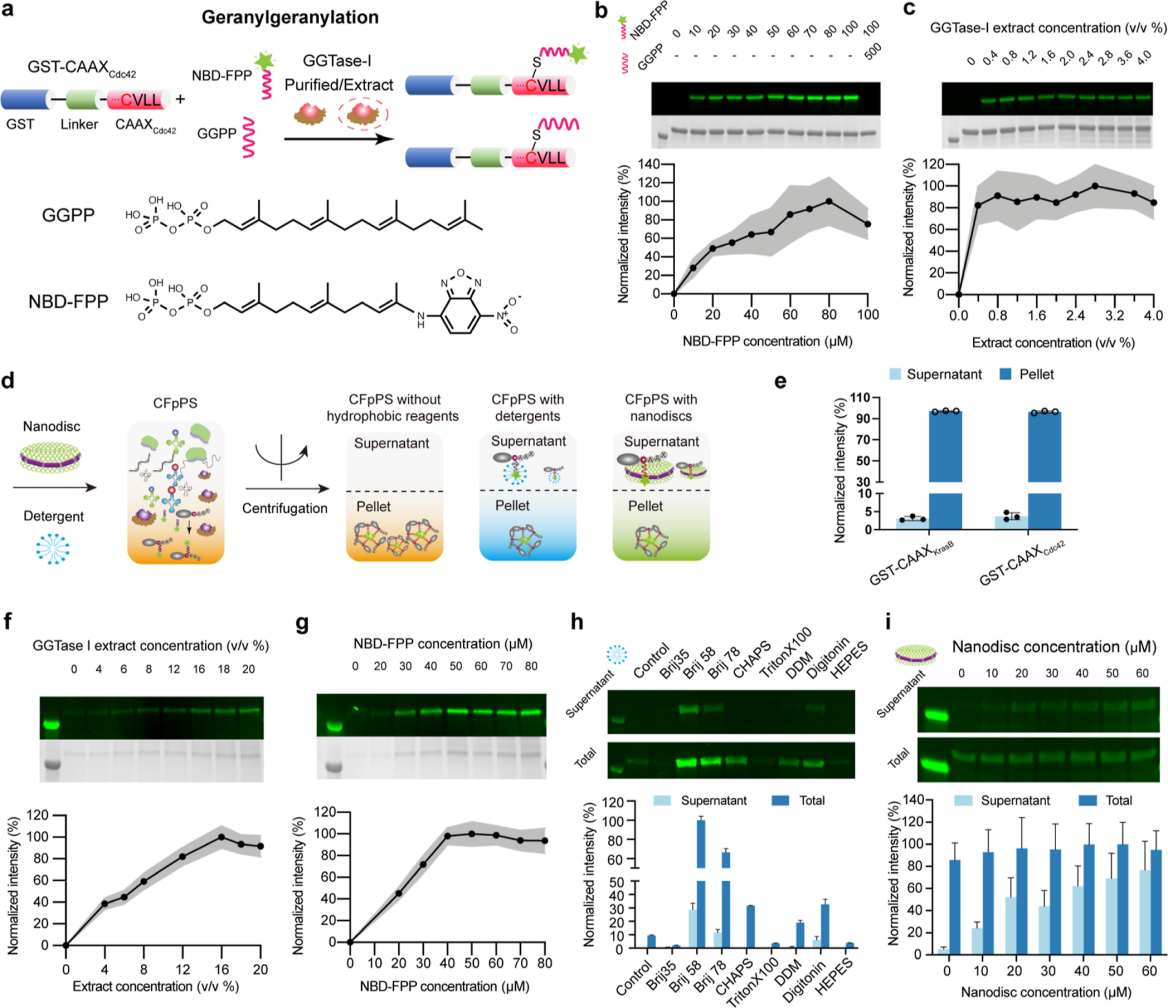
Establishment of CFpPS for geranylgeranylation. (a) Schematic illustration of the chimeric proteins GST-CAAX_Cdc42_ that are geranylgeranylated via purified GGTase-I- or GGTase-I-enriched extracts. (b) Titration of the NBD-FPP with purified GGTase-I using in-gel fluorescence. The last lane showed the competition assay performed by adding the unlabeled analogue—GGPP—at a concentration fivefold that of the highest tested for the NBD-modified analogue. Concentration (μM) of lipid donor in each reaction is stated above the corresponding gel lane. (c) Titration of GGTase-I-enriched extracts using in-gel fluorescence with10 μM GST-CAAX_Cdc42_ and 80 μM NBD-FPP. Extract concentration is shown as percentage volume of the GGTase-I-enriched extract included in the standard *E. coli* CFPS. (d) Schematic depicting the expression and solubilization of prenylated CAAX proteins in CFpPS systems with or without solubilizing additives. (e) Prenylated GST-CAAX_KrasB_ or GST-CAAX_Cdc42_ demonstrates low solubility after co-translational prenylation in CFpPS extracts lacking solubilizing additives. 20/80 μM NBD-GPP/FPP were used and prenylated proteins were measured using in-gel fluorescence in the supernatant and the pellet fractions after centrifugation at 20,000*g*. Measurements were normalized to the mean total protein amount in both the pellet and soluble fractions for each protein. Symbols represent intensity measured in three independent replicates. (f) Concentration optimization of the GGTase-I-enriched extract in the CFpPS system using in-gel fluorescence. Extract concentration is shown as percentage volume of the enriched extract included in the standard *E. coli* CFPS. (g) In-gel fluorescence analysis for optimizing the concentration of NBD-modified lipid donor in the CFpPS system. (h) Screening of detergents for soluble expression of geranylgeranylated GST-CAAX_Cdc42_. Respective control reactions were performed without any detergent. (i) Nanodisc titration for the soluble expression of GST-CAAX_Cdc42_ in the CFpPS system. Fluorescence intensities of the protein band for each fraction were measured through in-gel fluorescence. Each image (b,c,f,g) includes a representative gel imaged in fluorescence mode to visualize NBD (upper) and colorimetric mode to visualize Coomassie staining (lower). In all graphs, intensity is normalized to the highest average value measured in a dataset. In all graphs (b,c,f,g), mean values from three independent replicates are shown as black dots, while the gray shading represents standard deviation, *n* = 3.

Prenylation is catalyzed by enzymes known as prenyltransferases.^23^ Depending on whether the isoprenoid involved is a 15-carbon farnesyl group or a 20-carbon geranylgeranyl group, prenyltransferases are known as farnesyltransferase (FTase) or geranylgeranyltransferase (GGTase), respectively; the corresponding lipid substrates are farnesyl-diphosphate (FPP) and geranylgeranyl-diphosphate (GGPP), respectively. Among the four members of prenyltransferase,^24−26^ we selected FTase and GGTase-I as model enzymes (Figures S1–S3) since their enzymatic functions have been well-characterized before in vitro.^27^ Despite existing in vitro prenylation methods using purified enzymes^19^ or crude lysates^28^ as well as the in vivo approach to produce prenylated proteins,^29^ the unstable nature of target CAAX proteins pre- and post-prenylation still hampered the reconstitution of membrane switches based on proteins such as Cdc42. Although previously reported eukaryotic cell-free systems have succeeded in obtaining prenylated proteins,^30,31^ they either suffer from extremely low expression yields or low modification efficiency, thereby not meeting the requirements for designing more sophisticated membrane switches. Therefore, we selected the most productive cell-free system based on *E. coli* cell extracts^22,32^ and integrated the prenylation machinery to achieve both high expression yield and modification efficiency. To integrate the prenylation machinery into the bacterial CFPS system, we prepared extracts from *E. coli* cells overexpressing a prenyltransferase of interest (Figure S1). The resulting combinations of the standard bacterial CFPS system with prenyltransferase-enriched extracts will, henceforth, be referred to as the CFpPS system (Figure 1b). To validate the efficiency of CFpPS, we carried out prenylation with a fluorescently labeled lipid substrate^33^ (Figures 2a and S4a) and detected the prenylated protein on sodium dodecyl-sulfate polyacrylamide gel electrophoresis (SDS-PAGE) gels using in-gel fluorescence (Figure 1b). To serve as protein substrates for prenylation, we designed different chimeric proteins: The C-terminal amino acids of the small GTPases KrasB and Cdc42 were added to the C-terminus of the well-characterized glutathione S-transferase (GST) protein, connected by a rigid helical linker (Figures 2a, S4a and Materials and Methods for details). FTase catalyzed the prenylation of GST-CAAX_KrasB_ by NBD-GPP, whereas GGTase-I catalyzed prenylation of GST-CAAX_Cdc42_ with NBD-FPP. The combination of these model reaction components enabled us to optimize parameters for the CFpPS system, such as concentrations of isoprenoids and the ratio of the component extracts for subsequent experiments.

Before testing prenylation extracts, we examined whether the purified prenyltransferases FTase and GGTase-I could prenylate their purified protein substrates GST-CAAX_KrasB_ and GST-CAAX_Cdc42_, respectively (Figures 2b and S4b; Figures S2 and S3 for the purification of prenyltransferases). This enabled us to validate the in-gel fluorescence assay and to determine the effective concentrations of NBD-modified prenyl donors in a more controlled setting. For both reactions, at a fixed concentration of 10 μM protein substrate, the fluorescence intensity of the protein substrate’s band increased with ascending concentrations of its isoprenoid substrate, up to a certain concentration. Maximum activity was achieved at 20 μM isoprenoid concentration for farnesylation and 80 μM for geranylgeranylation. These were the concentrations chosen for subsequent tests with extracts. In both cases, prenylation output decreased marginally when isoprenoid concentration was further increased, possibly due to concentration-dependent aggregation of the isoprenoid substrates. Lastly, the protein band showed no fluorescence when natural isoprenoid substrates FPP and GGPP were included in the reactions at five times the highest concentration of their fluorescent analogues NBD-GPP and NBD-FPP, respectively (Figures 2b and S4b, right-most lanes in gels). This competition assay confirmed that read-outs of in-gel fluorescence were specific to the prenylation reactions of interest.

Next, we used in-gel fluorescence to verify that prenylation activity was intact in prenyltransferase-enriched cell extracts. For enriched extracts, competent *E. coli* cells were simultaneously transformed with plasmid vectors carrying the alpha and beta subunits of the prenyltransferase of interest (either FTase or GGTase-I; Figure 1b), and overexpression was induced using IPTG. After induction, crude extract was prepared using the standard S30 preparation procedure (see Materials and Methods for details). Using previously optimized concentrations of the fluorescent isoprenoids, the functionality of each prenylation extract was verified through in-gel fluorescence read-outs (Figures 2 and S4c). Prenylation outputs saturated at around 0.8% (v/v) for both farnesylation extracts and geranylgeranylation extracts, which roughly corresponds to 0.83 μM purified FTase and 1.22 μM GGTase-I (Figure S5). Although saturation concentrations were slightly different from the purified enzyme (0.4 μM FTase or 2 μM GGTase-I), the prenyltransferase-enriched cell extracts were fully compatible with prenylation reactions, which bypass tedious purification procedures and maintain high recovery rates.

Finally, to complete the one-pot protein synthesis and prenylation system, we allowed the chimeric substrates to be directly expressed in the prenylation extracts. As expected, the prenylated form of both GST-CAAX_KrasB_ and GST-CAAX_Cdc42_ ended up in the pellet fraction upon centrifugation since the isoprenoid group increased their hydrophobicity (Figure 2e). We thus resolved the pellet fraction on SDS-PAGE for subsequent in-gel fluorescence assays to titrate the composition of the CFpPS system. First, we optimized the ratio of prenyltransferase-enriched extract to standard CFPS extract to achieve a maximum prenylated protein. This screen was carried out at a fixed NBD-modified isoprenoid concentration of 30 μM (Figures 2f and S4d). Second, we used the extract ratios of maximum activity (2% v/v for FTase extract and 16% v/v for the GGTase-I extract) to titrate the NBD isoprenoid concentrations (Figures 2g and S4e). Unlike the reaction with defined components, the reaction in the CFpPS system saturated at higher concentrations of NBD-lipid donors (e.g., 80 μM instead of 20 μM NBD-GPP for farnesylation). In addition, more enriched extract was required to achieve the same prenylation output. This reduction in CFpPS efficiency when protein substrates were co-translationally prenylated could be due to multiple reasons. For instance, non-specific interactions with residual membrane vesicles from the extract^34,35^ could limit the availability of either the lipid donors or the prenyltransferases and hence lead to higher saturation concentrations of both substrates and enzymes.

### Soluble Expression of Prenylated Proteins

To solve the challenge of soluble expression of prenylated proteins, we select different hydrophobic reagents, including detergents and nanodiscs (Figures 1b and 2d). One major advantage of the CFpPS system is that solubilizing agents such as detergents or lipids can be introduced to co-translationally solubilize the prenylated CAAX-proteins. Absence of hydrophobic environments can lead to aggregation of the modified protein or unspecific binding to chromatography resins, leading to significant losses during protein purification.^29^ First, we tested detergents, which are often used in CFPS to improve the solubility of expressed membrane proteins.^36^ The compatibility of seven commonly used detergents was evaluated by determining the prenylation efficiency in the presence of the detergent using in-gel fluorescence (Figure S6). Next, these detergents were directly introduced in the CFpPS system to assess their ability to keep the prenylated protein soluble. After centrifugation, supernatant, pellet, and total protein fractions were collected and evaluated for the presence of the prenylated proteins (Figures 2h and S4f). Nearly all detergents tested showed little increase in the solubility of farnesylated GST-CAAX_KrasB_ (Figure S4f). In contrast, the solubility of the geranylgeranylated GST-CAAX_Cdc42_ increased more than 80-fold in the presence of certain detergents such as Brij58 (Figure 2h). Interestingly, the introduction of detergents could also improve the total prenylation efficiency for GGTase-I, with a nearly 10-fold increase in the presence of Brij 58 and 30% of the modified protein accounted for in the soluble fraction. This effect was likewise detected in the in vitro geranylgeranylation reaction; however, the improvement was small compared to that observed in the CFpPS system (Figure S6). It is possible that the improvement in modified protein fractions might be due to better solubility and availability of either the protein substrate or of the prenyl donor GGPP in the presence of detergents.

As an alternate solubilization strategy, particularly for farnesylation, we introduced nanodiscs to the CFpPS system. Nanodiscs are discoidal lipid bilayers stabilized by the presence of amphipathic protein belts and are thus closer mimics of the cell membrane^37^ (Materials and Methods for details). Negatively charged lipids were included in the nanodiscs to promote the association of prenylated proteins through the conserved polybasic regions of native CAAX proteins.^38^ Nanodiscs could in fact increase the soluble fraction of farnesylated GST-CAAX_KrasB_ up to sixfold, although there was a slight decrease in total modified proteins (Figure S4g). For geranylgeranylated GST-CAAX_Cdc42_, nanodiscs could increase the soluble fraction approximately 14 times compared to the control, corresponding to 72% of the total modified protein (Figure 2i). Notably, the total modified protein stayed relatively stable for geranylgeranylation. In summary, nanodiscs could greatly improve the solubility of farnesylated GST-CAAX_KrasB_, while either the detergent Brij 58 or nanodiscs promoted the solubility of geranylgeranylated GST-CAAX_Cdc42_.

Finally, we tested the performance of the optimized CFpPS system for expressing and solubilizing various representative CAAX proteins. The CFpPS system succeeded in expression and prenylation of not just chimeric proteins with CAAX sequences but also a selection of the native small GTPases; furthermore, all proteins except RhoA and RhoC could be produced in a soluble form, aided either by nanodiscs or by Brij 58 (Table S1, Figure S7). The effect of Brij 58 detergents in improving the total modified protein is shown in Cdc42 amidst all the RhoGTPase tested (Table S1, Figure S7). In addition to solubilizing reagents, the overall stability of the target protein can influence the efficiency of producing a prenylated protein. This intrinsic property of a protein can lead to starkly different outputs for different proteins under the same solubilization conditions (see Figure S7 and Table S1). Hence, the optimum solubilization conditions would be target-dependent and require empirical determination for each protein of interest.^39−41^

### Direct Targeting of CFpPS-Produced Chimeric Protein to Biomimetic Membranes

**Figure 3:**
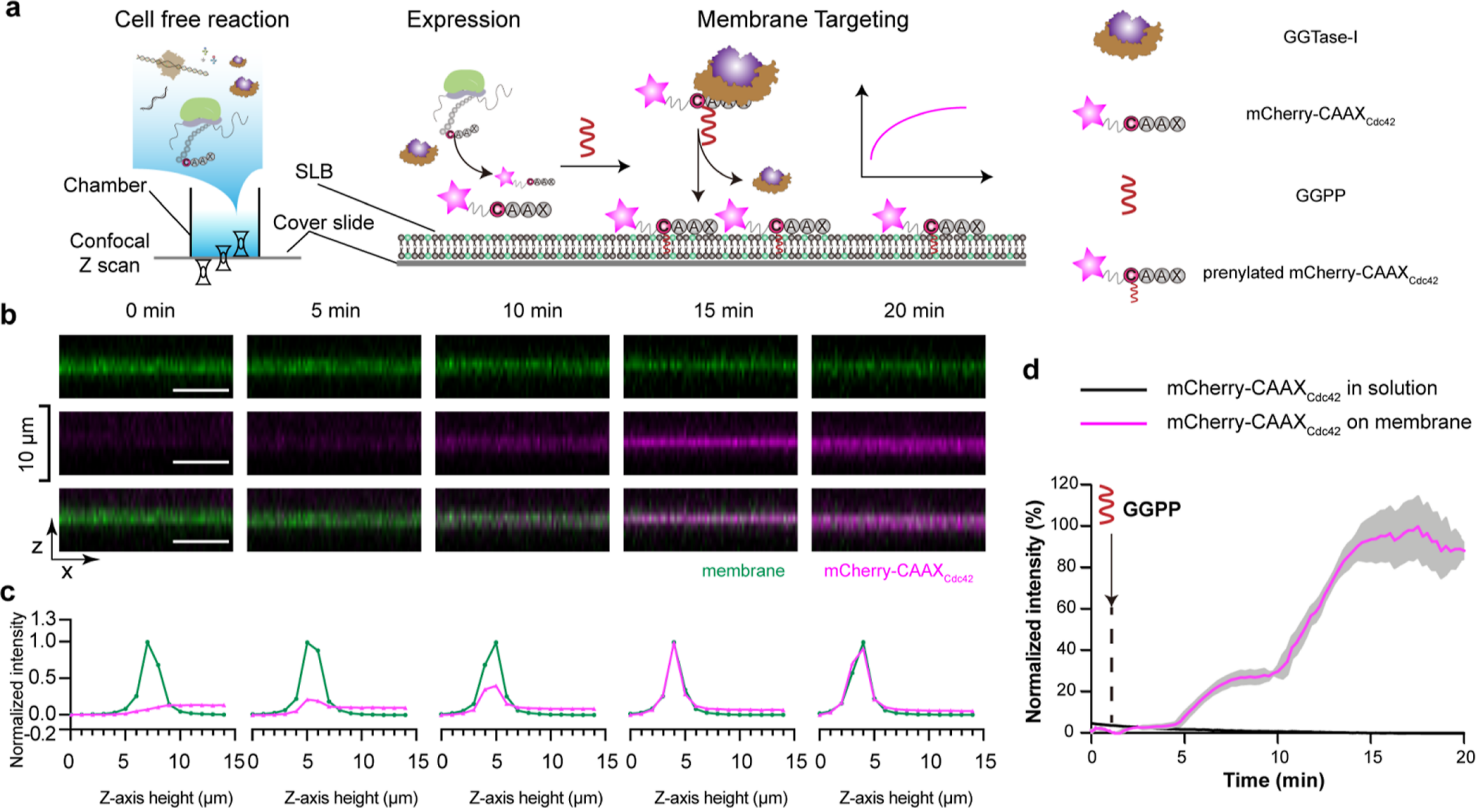
Prenylated mCherry-CAAX_Cdc42_ produced using the CFpPS system binds to biomimetic membranes. (a) Schematic illustration of the membrane targeting of mCherry-CAAX_Cdc42_, as studied on SLBs using confocal microscopy. (b) Orthogonal views of the SLB membrane (upper, green), mCherry-CAAX_Cdc42_ (middle, magenta), and a merge of both channels (lower) at different time points after prenylation was initiated in the CFpPS reaction by adding GGPP. The SLB composition is 80% DOPC, 19.95% DOPS, and 0.05% Atto-488 PE. All scale bars are 10 μm. (c) Normalized intensities of corresponding images from (b). Intensities of mCherry-Cdc42 were normalized to maximum and minimum intensities recorded in the *z*-stack during the time-lapsed experiments; intensities of the membrane channel were normalized to maximum and minimum intensities recorded in the *z*-stack at each time point. (d) Time series of mCherry-CAAX_Cdc42_ intensity on the membrane (pink) and in solution (black). Intensities were normalized to maximum and minimum intensities measured during a time-lapse experiment. Solid lines represent the mean intensity measured over a 75-pixel by 75-pixel region, and gray shading represents the standard deviation. Data are representative of three independent replicates.

To test whether prenylated protein produced through CFpPS shows membrane interaction, we carried out the reaction of fluorescent chimeric protein—mCherry-CAAX_Cdc42_ via CFpPS on top of a planar SLB and monitored the membrane-targeting process using confocal microscopy (Figure 3a). Localization of the protein substrate could be followed using the fluorescence of mCherry, whereas a small fraction of the fluorescently labeled lipid Atto-488 PE was included in the SLB to visualize the membrane. The lipid composition of the SLB was identical to the one used previously for nanodiscs. All components of the CFpPS reaction, except the prenyl donor GGPP, were mixed in a chamber with a preformed SLB. GGPP was withheld to trigger the prenylation process. As expected, upon addition of GGPP, we observed an increase in mCherry fluorescence on the SLB, indicating that the geranylgeranylated mCherry-CAAX_Cdc42_ was attaching to the membrane. Orthogonal sections (Figure 3b) through the confocal stack as well as measured *Z*-axis intensity profiles (Figure 3c) show that mCherry fluorescence is colocalized with the membrane. The mCherry signal continued to increase until it saturated roughly 15 min after the addition of GGPP (Figure 3d). We thus demonstrated that geranylgeranylation is sufficient to target a chimeric protein to the membrane. In contrast to farnesylated proteins which may require additional carboxyl methylation for stable membrane association,^29^ additional modifications are less significant for a geranylgeranylated protein such as Cdc42.^20^ Thus, using CFpPS-assisted geranylgeranylation, membrane targeting can be introduced to any soluble protein simply by including a CAAX motif at its C-terminus.

### Reversible Membrane Targeting of Cdc42 Synthesized by the One-Pot CFpPS System

**Figure 4:**
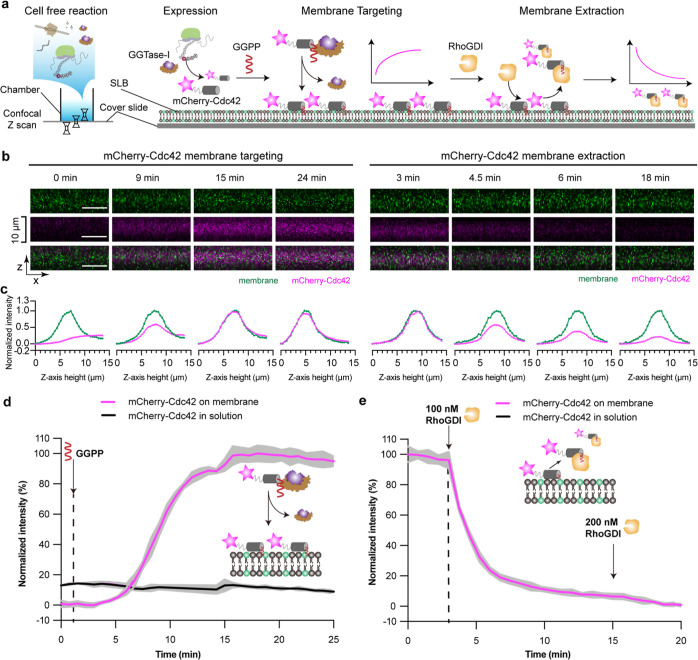
Reconstitution of the reversible membrane-binding of mCherry-Cdc42 produced by the CFpPS system. (a) Schematic illustration of the reconstitution of Cdc42’s membrane targeting and RhoGDI-dependent membrane extraction on SLBs, as visualized by confocal microscopy. (b) Representative images of orthogonal views of the SLB membrane (upper, green), mCherry-Cdc42 (middle, magenta), and a merge of both channels (lower) at different time points. Time was measured from the addition of GGPP during membrane targeting and from the initiation of time-lapse imaging for the extractions process. All scale bars are 10 μm. (c) Normalized intensities of corresponding images from (b). Intensities of mCherry-Cdc42 were normalized to maximum and minimum intensities recorded in the *z*-stack during the time-lapsed experiments; intensities of the membrane channel were normalized to maximum and minimum intensities recorded in the *z*-stack at each time point. During extraction, the same normalization was performed for the membrane channel; while the intensities of mCherry-Cdc42 were normalized to the maximum intensity recorded in the *z*-stack at 3 min before the addition of RhoGDI. (d,e) Time series of mCherry-Cdc42 intensity on the membrane (pink) and in solution (black) during membrane targeting (d) and membrane extraction (e). Intensities were normalized to maximum and minimum intensities measured during a time-lapse experiment. Solid lines represent the mean intensity measured over a 75-pixel by 75-pixel region, and gray shading represents the standard deviation. Data are representative of three independent replicates. The lipid composition of SLBs used in this figure is 80% DOPC, 19.95% DOPS, and 0.05% Atto-488 PE.

Having demonstrated the membrane targeting of chimeric mCherry-CAAXCdc42, we moved to the final step of reconstituting a Cdc42-based membrane switch. Here, instead of the short CAAX motif, we designed a new chimeric construct, where full length Cdc42 was fused to the C-terminus of mCherry (Figure 4a). Prenylated Cdc42 should form a complex with its interacting partner RhoGDI and subsequently dissociate from the membrane. Using the same one-pot experimental setup, the localization of the chimeric protein could be visualized by mCherry fluorescence and Atto-488 labeled lipids (Figure 4a). The membrane targeting process was triggered via the addition of GGPP, while the membrane extraction was primed by addition of RhoGDI (Figure 4a,d,e). Similar to mCherry-CAAX_Cdc42_, mCherry-Cdc42 intensity on the membrane increased until saturation at roughly 15 min after the addition of GGPP (Figure 4d). Orthogonal view (Figure 4b) and *Z*-axis intensity profiles (Figure 4c) both show that mCherry-Cdc42 colocalizes with the membrane marker at that time point. Prenylated Cdc42 is known to bind to negatively charged lipids due to the hyper-variable region upstream of the CAAX motif, containing multiple positively charged amino acid residues.^42^ To verify the specificity of membrane interaction, we used both neutral and negatively charged lipid to form the SLB. As expected, prenylation-driven membrane binding was significantly better on negatively charged membranes (Figure S8).

To demonstrate the reversibility of mCherry-Cdc42’s membrane interaction, we then added RhoGDI to trigger the extraction of Cdc42 from the membrane.^43^ Upon addition of RhoGDI, mCherry fluorescence sharply decreased on the membrane, indicating that membrane-bound mCherry-Cdc42 was extracted from the SLB (Figure 4b,c,e). After more than two-thirds of the membrane-bound Cdc42 had been extracted, introducing additional RhoGDI did not lead to further decrease in mCherry fluorescence on the membrane, indicating that the remaining Cdc42 may not be extractable (Figure 4e). Unlike the full-length Cdc42, the chimeric construct mCherry-CAAX_Cdc42_ could not be extracted by RhoGDI after binding to the SLB, confirming that the extraction process we saw for the full-length Cdc42 results from specific protein–protein interactions (Figure S9). Unlike for Kras,^29^ post-prenylation modification steps of Cdc42, such as cleavage of last three amino acids and carboxyl methylation, influence neither the membrane binding nor the interaction with its regulator protein RhoGDI.^44^ Altogether, the CFpPS system enabled reconstitution of a reversible membrane switch with a minimal two-component system, consisting of full-length Cdc42 and RhoGDI on an SLB in a one-pot configuration—a process of great functional relevance to the polarity regulator Cdc42.^12,20^ Furthermore, the chimeric design of mCherry-Cdc42 demonstrates that this reversible membrane switch system could be leveraged to target soluble proteins (mCherry, in this case) to the membrane. As an additional advantage of our setup, CFpPS could be carried out directly atop an SLB, allowing easy introduction of additional protein regulators into the same chamber. These proteins could be added in a purified form when their concentration is of key relevance. Alternatively, proteins of interest could be introduced as plasmids, which get translated alongside Cdc42 by CFpPS, thus opening vast possibilities for rapid and high-throughput testing of chimeric proteins. Last but not the least, considering the compatibility of the established CFpPS setup with model membranes (i.e., SLB) and light microscopy, it offers a promising in vitro platform for the rigorous study of complex multi-protein processes, such as polarization.

## Conclusions

In this study, we have successfully established one-pot expression and membrane targeting of proteins by leveraging CAAX prenylation in an in vitro cell-free system. We have achieved this by integrating prenylation machineries corresponding to different lipid substrates into the standard CFPS system. The resulting CFpPS enabled the efficient expression and co-translational prenylation and solubilization of both synthetic constructs with exogenous CAAX motifs and natural CAAX proteins. Moreover, such membrane targeting could be timed by withholding the prenyl donor, resulting in switch-like membrane recruitment, which can be highly beneficial for the study of signaling cascades. This property offers an added advantage over previously used reconstitution strategies such as including nickelated or biotinylated lipids in the membrane and adding a His tag or Strep tag, respectively, to the protein of interest.^9,45^ In addition, this one-pot CFpPS is conducive to fast assessment by light microscopy on biomimetic model membranes, thus bypassing the challenges of expression, purification, and delivery of prenylated proteins which have been observed in traditional in vivo expression or when using limited prenylation machinery from eukaryotic cell-free systems.^28−31^

More importantly, we realized the feature of reversible membrane binding by sequentially introducing an interaction protein RhoGDI to the membrane-bound Cdc42 protein in the same one-pot CFpPS setup, which, altogether, achieved the reconstitution of a reversible membrane switch with only two protein components. The established reversible membrane-targeting system not only offers an in vitro biomimetic setup to investigate the mechanistic role of the membrane switch in Cdc42-based polarization but also provides a way to confer reversible switch-like properties to other proteins by fusion to Cdc42. Taken together, we postulate that the general design of reversible membrane switches based on CFpPS holds great potential for studying the elaborate protein interaction networks of small GTPases using bottom-up synthetic biology.

## Materials and Methods

### Bacterial Strains and Plasmids

A list of all plasmids generated and used in this study can be found in the Supporting Information (Table S2). Protein and gene sequences as well as cloning procedures are provided in the “Gene and Protein Sequences” section in the Supporting Information.

### Cloning Methods

*E. coli* One Shot TOP10 (Invitrogen, Thermo Fisher Scientific, Waltham, USA) cells were used for the propagation of all plasmids. All gene sequences created in this study were synthesized by Eurofins Genomics Europe in cloning vectors. Each gene was then amplified and subcloned into the expression vector using sequence- and ligation-independent cloning methods.^46^ For fusion constructs with the C-terminal sequence of CAAX proteins, a long rigid linker^47^ (protein sequences in italics) was used, such as GST-KrasB, GST-Cdc42, and mCherry-Cdc42_CAAX_. The 3C protease recognition sequences are highlighted with underlines.

### Protein Expression and Purification

Purifications of FTase and GGTase-I were performed according to previous protocols.^33,48^ Briefly, a single colony of *E. coli* Rosetta carrying both plasmids for both the α and the β subunits was grown overnight at 30 °C in 50 mL of Luria Bertani (LB) medium (10 g L^–1^ tryptone, 5 g L^–1^ yeast extract, and 5 g L^–1^ NaCl) containing 50 μg/mL carbenicillin (CA), 50 μg/mL kanamycin (KAN), and 37 μg/mL chloramphenicol (CHL). Then, 500 mL terrific broth (TB) medium [20 g L^–1^ tryptone, 24 g L^–1^ yeast extract, 0.4% (v/v %) glycerol, and 10% (v/v %) phosphate buffer (0.17 M KH_2_PO_4_/0.72 M K_2_HPO_4_)], including CA/KAN/CHL, was inoculated with 10 mL of overnight culture and grown at 37 °C until Abs_600_ was 0.9–1.0. Protein expression was induced by adding 0.4 mM IPTG and further incubation at 37 °C for 4 h (see Figure S1). Cells were harvested by centrifugation at 8000*g* at 4 °C, followed by two washes with phosphate-buffered saline buffer. The resulting cell pellet was resuspended in 30 mL of lysis buffer [50 mM phosphate buffer pH 8.0, 0.3 M NaCl, 1 mM TCEP, and 0.1 mM phenylmethylsulfonyl fluoride (PMSF)], and cells were disrupted via a single pass through a pre-cooled French Press at 17,000 psi prior to 30 min of incubation on ice with 10 U/mL benzonase nuclease. The lysate was then centrifuged at 20,000*g* for 30 min. The supernatant was filtered through a 0.22 μm PVDF membrane and loaded onto a 5 mL HiTrap GST column via an ÄKTA pure protein system (Cytiva). Eluted peak fractions were pooled and diluted 10-fold with anion exchange buffer (50 mM phosphate buffer pH 8.0 and 1 mM TCEP). The resulting mixture was loaded onto an anion exchange Hitrap Q FF column (Cytiva) and washed with 50 column volumes of buffer (50 mM phosphate buffer pH 8.0 and 1 mM TCEP). FTase or GGTase-I were eluted through the linear increase of NaCl to a final concentration of 1 M. Peak fractions were collected and dialyzed against 5 L of dialysis buffer (50 mM HEPES, pH 7.2, 100 mM NaCl, and 5 mM DTT). Dialyzed samples were further concentrated to 10 mg mL^–1^ using an Amicon Ultra-15 Centrifugal Filter Unit (Merck Millipore). The final concentration of glycerol was adjusted to 50% (v/v %) for storage at −80 °C.

GST-CAAX_KrasB_, GST-CAAX_Cdc42_, and RhoGDI were expressed in *E. coli* BL21 (DE3) in TB medium following the same protocol as the FTase and GGTase-I purification, except that the IPTG concentration was increased to 1 mM and protein expression was performed via overnight incubation at 16 °C. GST-CAAX_KrasB_ and GST-CAAX_Cdc42_ were stored in storage buffer (50 mM HEPES, pH 7.8, 150 mM NaCl, 1 mM TECP, and 15% v/v glycerol) and flash-frozen in liquid nitrogen prior to their storage at −80 °C. For purification of RhoGDI via a Ni-NTA column, the following buffers were used: lysis buffer (50 mM Tris-HCl, pH 7.4, 300 mM NaCl, 2 mM TCEP, 5 mM MgCl_2_, and 0.1 mM PMSF), wash buffer (50 mM Tris-HCl pH 7.4, 300 mM NaCl, 2 mM TCEP, 5 mM MgCl_2_, and10 mM imidazole), and elution buffer (50 mM Tris-HCl pH 7.4, 300 mM NaCl, 2 mM TCEP, 5 mM MgCl_2_, and 250 mM imidazole). RhoGDI was further purified through gel filtration on a Superdex 75 size exclusion column (storage buffer: 50 mM Tris-HCl pH 7.4, 300 mM NaCl, 1 mM TCEP, and 5 mM MgCl_2_), and protein fractions were pooled and concentrated prior to storage in single-use aliquots at −80 °C.

mCherry-Cdc42 was expressed as outlined above. Purification was performed using a HisTrap column and a Superdex 75 column, and the protein was stored in storage buffer (20 mM HEPES pH 7.8, 150 mM NaCl, 2 mM MgCl_2_, 0.1 mM GDP, 0.5 mM TCEP, and 10% v/v glycerol) at −80 °C as single-use aliquots.

### Preparation of the S30 Extract

The standard S30 extract for CFPS was prepared according to previously published protocols.^40,49^ Briefly, *E. coli* BL21 (DE3) cells were grown until the mid-log growth phase (Abs_600_ around 3.0) in 2 L baffled Erlenmeyer flasks. Cells were fast-chilled for 10 min under ice cold water and harvested via centrifugation at 8000*g* for 15 min. Cell pellets were washed three times with pre-cooled S30 A buffer (10 mM Tris-acetate pH 8.2, 14 mM Mg(OAc)_2_, 60 mM KCl, and 6 mM β-mercaptoethanol) and resuspended with 110% (v/w %) volume S30 B buffer (10 mM Tris-acetate pH 8.2, 14 mM Mg(OAc)_2_, 60 mM KCl, 1 mM DTT, and 1 mM PMSF). The resuspended cells were disrupted via a single pass through a French Press at 17,000 psi. The resulting lysates were clarified via two rounds of centrifugation at 30,000*g*. The supernatant was mixed with 0.3 volume of pre-incubation buffer (300 mM Tris-acetate pH 7.6, 10 mM Mg(OAc)_2_, 10 mM ATP, 80 mM phosphoenolpyruvat, 5 mM DTT, 40 μM each of the 20 amino acids, and 8 U mL^–1^ pyruvate kinase) and incubated at 37 °C for 80 min. Samples were then dialyzed for 2 h against a 100-fold volume of S30 C buffer (10 mM Tris-acetate pH 8.2, 14 mM Mg(OAc)_2_, 60 mM KOAc, and 0.5 mM DTT) and again overnight at 4 °C. The dialyzed samples were centrifuged at 30,000*g* for 30 min; the supernatant was collected into small aliquots, frozen with liquid nitrogen, and stored at −80 °C until further usage. For the prenyltransferase-enriched extract, double-transformed cells were induced at an Abs_600_ of 1.0 with 0.4 mM IPTG for 3 h (final Abs_600_ was around 4.0). All further steps were identical to the outlined standard S30 extract preparation.

### Preparation of T7 Polymerase

Preparation of T7 polymerase was performed as described previously.^40^*E. coli* strain BL21 (DE3) Star was transformed with plasmid pAR1219^50^ carrying the T7 polymerase gene. 1 L of LB medium with antibiotics was inoculated with an overnight culture at 1:100 ratio. Cells were grown on a shaker at 37 °C until Abs_600_ reached 0.6–0.8. T7 polymerase production was induced by addition of IPTG (1 mM final concentration in media). Cells were cultured for 5 h and harvested by centrifugation at 8000*g* for 15 min at 4 °C. Cell pellets were resuspended in 30 mL of T7 buffer A (30 mM Tris-HCl, pH 8.0, 50 mM NaCl, 1 mM EDTA, 10 mM β-mercaptoethanol, and 5% glycerol) and disrupted via a single pass through a French Press at 15,000 psi. Then, the cell lysate was clarified via centrifugation at 20,000*g* for 30 min at 4 °C, and the supernatant was adjusted to a final concentration of 4% (w/v %) streptomycin sulfate. The sample was then centrifuged at 20,000*g* for 30 min at 4 °C. The resulting supernatant was filtered and loaded onto a 40 mL Q-Sepharose column pre-equilibrated with T7 buffer B [30 mM Tris-HCl, pH 8.0, 50 mM NaCl, 1 mM EDTA, 10 mM β-mercaptoethanol, and 5% (v/v %) glycerol] and washed extensively with T7 buffer B after loading. T7 polymerase was then eluted using a linear gradient of 50–500 mM NaCl and 10 column volumes of T7 buffer B. Collected fractions were analyzed by SDS-PAGE. Fractions containing T7 polymerase (a predominant band around 90 kDa) were pooled and subsequently dialyzed against T7 buffer C [10 mM K_2_HPO_4_/KH_2_PO_4_, pH 8.0, 10 mM NaCl, 0.5 mM EDTA, 1 mM DTT, and 5% (v/v %) glycerol] overnight. Glycerol was added to a final concentration of 10% (v/v %), and the protein was concentrated to 3–4 mg mL^–1^ by ultrafiltration. Additional glycerol was added to a final concentration of 50% (v/v %); single-use aliquots were flash-frozen using liquid nitrogen and stored at −80 °C.

### In Vitro Prenylation Assay and In-Gel Fluorescence Analysis

For in vitro protein prenylation, reaction mixtures (volume 20 μL) were composed of: 10 μM CAAX protein, 0.4 μM FTase or 2 μM GGTase-I, and the respective indicated concentrations of NBD-GPP or NBD-FPP in prenylation buffer (50 mM HEPES pH 8.0, 300 mM NaCl, 20 μM ZnSO_4_, 2 mM MgCl_2_, 0.5 mM TCEP, and 100 μM GDP). Reaction mixtures were incubated for 2 h at 25 °C and quenched by adding 10 μL of 4× Laemmli sample buffer (Bio-Rad). Samples were boiled at 95 °C for 5 min and each 8 μL was loaded onto 12% SDS-PAGE gels. Prenylated protein bands were visualized in gel using an Amersham Imager 600RGB (Cytiva) [excitation light: blue epi light (460 nm) and emission filters: Cy2 (525BP20)]. After fluorescent imaging, gels were stained with Instant Blue (Expedeon) and scanned. For competition assays, natural prenyl-donors FPP or GGPP were additionally introduced beside the corresponding NBD analogues. Fluorescent images and Coomassie-stained images were analyzed by Fiji.^51^ Fluorescence intensities were calibrated by densitometry from the respective Coomassie-stained gel images to reduce loading error.

### Cell-Free Prenylated Protein Synthesis

Cell-free protein synthesis reactions were prepared according to protocols previously published by us and Kigawa et al.^40,49^ In brief, a typical CFPS reaction contained 55 mM HEPES-KOH buffer (pH 7.5), 1.7 mM DTT, 1.2 mM ATP (pH 7.0), 0.8 mM each of CTP (pH 7.0), GTP (pH 7.0), and UTP (pH 7.0), 80 mM creatine phosphate, 80 μg mL^–1^ creatine kinase, 2.0% (v/v %) PEG-8000, 0.65 mM 3,5-cyclic AMP, 68 μM folinic acid, 170 μg mL^–1^*E. coli* total tRNA, 200–250 mM potassium glutamate, 27.5 mM ammonium acetate, 15–20 mM magnesium acetate, 2.0 mM of each of the 20 amino acids, 10 μg mL^–1^ T7 polymerase (prepared according to above protocol), 30% (v/v %) S30 extract, and 15 ng μL^–1^ plasmid template. A typical reaction volume was 50 μL, and the reaction mixture was incubated at 30 °C for 2 h. CFpPS reactions contained prenyltransferase-enriched extract and the corresponding prenyl-lipid donor. Other additives such as nanodiscs (see section “Nanodiscs Preparation”) and detergents were added at respectively indicated concentrations to the cell-free reactions.

### Nanodisc Preparation

The preparation of nanodiscs was performed according to previous protocols.^52^ In brief, the MSP1E3D1 protein was purified via a Ni-NTA column. After elution, the protein-containing fractions were pooled with 10% glycerol (v/v) to prevent precipitation and were dialyzed overnight against the buffer [40 mM Tris-HCl, pH 8.0, 300 mM NaCl, and 10% (v/v %) glycerol], including a buffer exchange after 2 h. The resulting protein was centrifuged at 20,000*g* for 15 min to remove precipitated proteins. The samples were then aliquoted, frozen in liquid nitrogen, and stored at −80 °C until further usage. The nanodiscs used in this study were assembled via mixing of the following reagents: 25 μM MSP1E3D1, 1.6 mM DOPC (50 mM stock dissolved in 300 mM sodium cholate) (Avanti Polar lipids, Inc.), 0.4 mM DOPS (50 mM stock dissolved in 300 mM sodium cholate), and 0.1% (v/v %) *n*-dodecylphosphocholine in buffer (40 mM Tris-HCl, pH 8.0, and 300 mM NaCl). The mixture was incubated for 1 h at room temperature. Nanodisc-assembly was achieved by dialysis (1:500 volume ratio, buffer: 10 mM Tris-HCl, pH 8.0, 100 mM NaCl) using a Slide-A-Lyzer (Thermo Fisher Science) at room temperature for 12 h. The sample was again dialyzed for 24 h at 4 °C and centrifuged for 20 min at 20,000*g*. The supernatant was collected and concentrated using an Amicon filter unit (10 kDa, MWCO, Millipore). The final concentration of nanodiscs should be above 0.5 mM, which corresponds to 1 mM MSP1E3D1. Concentrated nanodiscs were aliquoted, flash-frozen in liquid nitrogen, and stored in −80 °C.

### SLB Formation

Preparation of glass coverslips (Menzel #1.5, 24 × 24 mm) and reaction chambers were performed according to previous protocols^9,53^ and are described in detail in the following section. SLBs were formed through fusion of small unilamellar vesicles (SUVs) on the preformed reaction reservoir. SUVs were prepared as follows: 80 mol % DOPC (Avanti Polar Lipids, Inc.), 19.95 mol % DOPS (Avanti Polar Lipids, Inc.), and 0.05 mol % ATTO488-DOPE (ATTO-TEC GmbH) were dissolved, mixed in chloroform, dried under a gentle stream of nitrogen, and transferred to a vacuum chamber for 1 h. Then, the dried lipid film was rehydrated in SLB buffer A (50 mM Tris-HCl pH 7.5, 150 mM KCl, and 5 mM MgCl_2_) to reach a final lipid concentration of 4 mg mL^–1^. Resulting samples were further vortexed and sonicated (bath sonicator, Branson) until they appeared clear. SLBs were prepared according to previously published protocols.^53^ Briefly, SUVs (4 mg mL^–1^) were diluted with 130 μL of SLB buffer A, and 75 μL of the suspension was transferred to the preformed reaction chambers and incubated on a heat block at 37 °C for 1 min. 150 μL of SLB buffer A was added into the chamber and incubated for further 2 min. The chamber was washed with 2 mL of SLB buffer B (SLB buffer A without MgCl_2_) prior to a buffer-exchange to either the prenylation buffer with 0.4% (w/v %) BSA (Sigma) for in vitro prenylation reactions or the S30C buffer with BSA for CFpPS reactions, leaving 100 μL of buffer inside the chamber to prevent drying of the formed SLBs. All used buffers had to be pre-warmed to avoid temperature fluctuations.

### Preparation of SLB Chambers

#### Cleaning of Coverslips

24 × 24 mm #1.5 coverslips (Menzel) were piranha-cleaned by adding 7 drops of sulfuric acid and two drops of 50% hydrogen peroxide to the center of each coverslip. The reaction was incubated on the coverslips for at least 45 min before thoroughly rinsing with ultrapure water.

#### Assembly of the Reconstitution Chamber

The reaction chamber was formed by attaching a cut 0.5 mL microfuge tube onto cleaned coverslips using optical glue (Norland Optical Adhesive 68, Norland Products) that was cured under a UV lamp (365 nm) for 10 min.

#### Reconstitution of CAAX-Protein Membrane Targeting and Extraction Processes

CFpPS reactions were composed as indicated above using corresponding plasmids without the GGPP substrate. 95 μL of the reaction mixtures was then transferred onto the SLB reservoir, and the chambers were set up on the confocal microscope equipped with a temperature-controlling system (ibidi heating system, universal fit chamber). Reactions were then started by adding 5 μL of GGPP to reach a final concentration of 10 μM. After the targeting process was finished, the SLBs were washed with prenylation buffer and RhoGDI was added to reach final concentrations of 100 and 200 nM.

#### Microscopy and Image Analysis

Imaging was performed on a Zeiss LSM780/LSM800 confocal laser scanning microscope using a Zeiss C-Apochromat 40×/1.20 water-immersion objective (Carl Zeiss AG, Oberkochen, Germany). A built-in definite focus was applied for imaging the time-series experiments. ATTO-488 (membrane-dye) was excited using the 488 nm laser, and mCherry fusion proteins were excited using the 594 nm laser. For multicolor imaging, images for each channel were acquired sequentially to prevent bleed-through. The resolution was set up to 512 × 512 pixels. To visualize the membrane localization, *z*-stack images (perpendicular to the plane of the SLB) were obtained with time intervals of 45 s. For control experiments using purified proteins, the temperature was kept constant at 25 °C, while for CFpPS reaction on SLBs, the temperature was maintained at 30 °C.

For analysis of membrane targeting, time series of *z*-stacks were processed using a custom Fiji macro (SI code). At each time point, the macro selected the membrane slice in the *z*-stack as the one with maximum mean intensity in the membrane channel and compiled the mean intensity of the corresponding mCherry channel into a new file. The resulting fluorescence intensities were then plotted over time. The average intensity of the last slice into the solution was used to represent the intensity from the solution during the membrane targeting process. For membrane extraction, the intensities were calculated as the mean intensities of the brightest slice at each time point without further calibration. Three individual replicates were performed per experimental condition.

## Supporting Information Available

Additional experimental, protein constructs, and system performance data; gene and protein sequences; and Fiji macro code (PDF)
