# Supplementary material for: Reconstitution of a Reversible Membrane Switch via Prenylation by One-Pot Cell-Free Expression
Source: ACS Synth Biol. 2022 Nov 29;12(1):108–19. doi: 10.1021/acssynbio.2c00406 (PMC9872162; doi:10.1021/acssynbio.2c00406)
Supplement: Supplementary file 1 — sb2c00406_si_001.pdf [file sb2c00406_si_001.pdf]

Supporting Information

Reconstitution of a reversible Membrane Switch via Prenylation by One-pot Cell Free Expression

Lei Kai[1,2,*] Sonal[1,3], Tamara Heermann[1], Petra Schwille[1*]

Affiliations:
1. Department of Cellular and Molecular Biophysics, Max Planck Institute of Biochemistry, D-82152 Martinsried, Germany
2. School of Life Sciences, Jiangsu Normal University, Shanghai Road 101, 221116 Xuzhou, P.R. China
3. Biosciences Division, University College London, Gower Street, London WC1E 6BT, UK

* corresponding authors:

Prof. Petra Schwille
ORCID: 0000-0002-6106-4847
schwille@biochem.mpg.de
Phone: +49 89 8578-2900
Fax: +49 89 8578-2903

Prof. Lei Kai
ORCID: 0000-0003-0879-7918
lkai@jsnu.edu.cn
Phone: +86 15852001351

Supplementary Figures

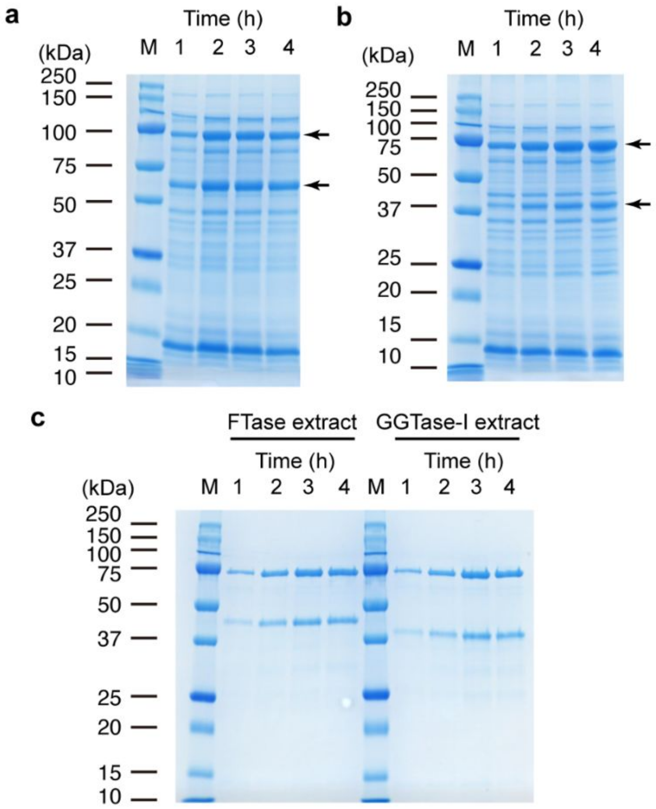

Figure S1. Screening of induction time for the preparation of prenyltransferase-enriched extracts. Coomassie-stained SDS-PAGE gels of clarified FTase **a)** and GGTase-I **b)** enriched cell lysates for varying time-points (1, 2, 3 or 4 h) after induction of expression. **c)** Coomassie-stained gel of the IMAC-purified prenyltransferase-enriched lysates after induction of expression. Numbers above each lane represent the induction time in hours. Black arrows highlight the band corresponding to the α and β subunits of the prenyltransferase.

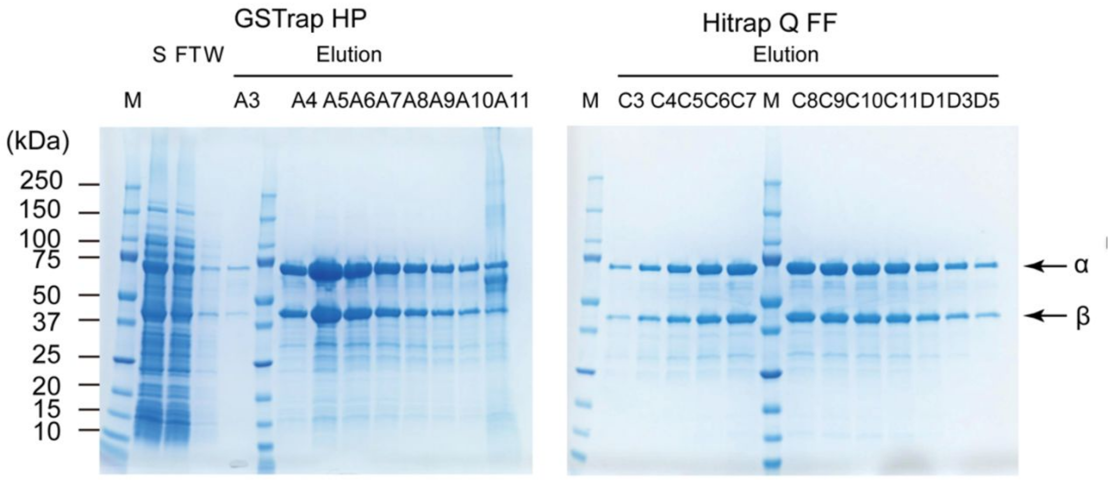

Figure S2. Purification of the prenyltransferase FTase. Coomassie-stained gels of collected fractions from the purification via a GSTrap HP column (left panel), followed by a Hitrap Q FF column (right panel). Lanes are titled with the fraction number obtained during elution. S indicates the supernatant, FT the flow-through, W the washing step and M denotes the protein marker. Black arrows indicate the bands that correspond to the α and β subunit of the FTase.

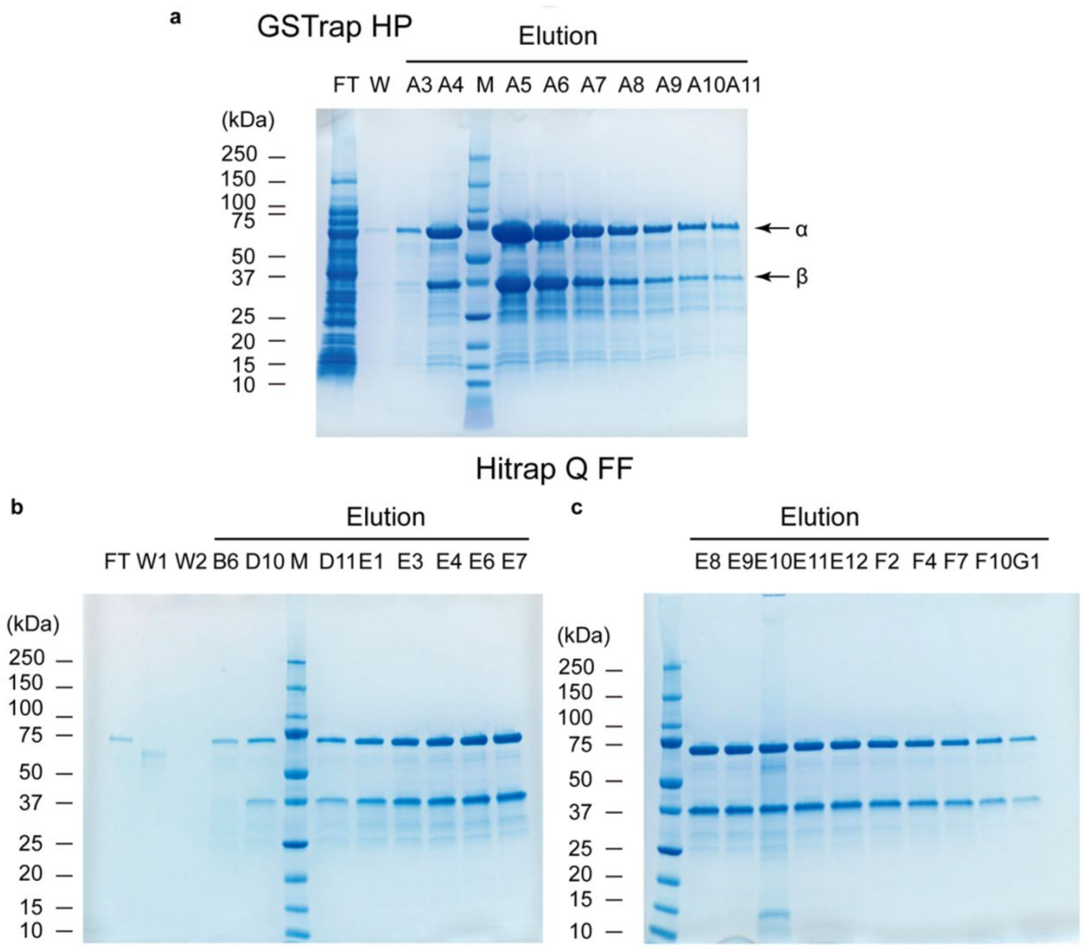

Figure S3. Purification of the prenyltransferase GGTase-I. Coomassie-stained gels of fractions collected from the purification via a GSTrap HP column **a)**, followed by a Hitrap Q FF column **b-c)**. Lanes are titled with the fraction number obtained during elution. FT indicates the flow-through and W, W1 and W2 the washing steps. M denotes the protein marker in all gels. Black arrows indicate the bands corresponding to the α and β subunit of the GGTase-I.

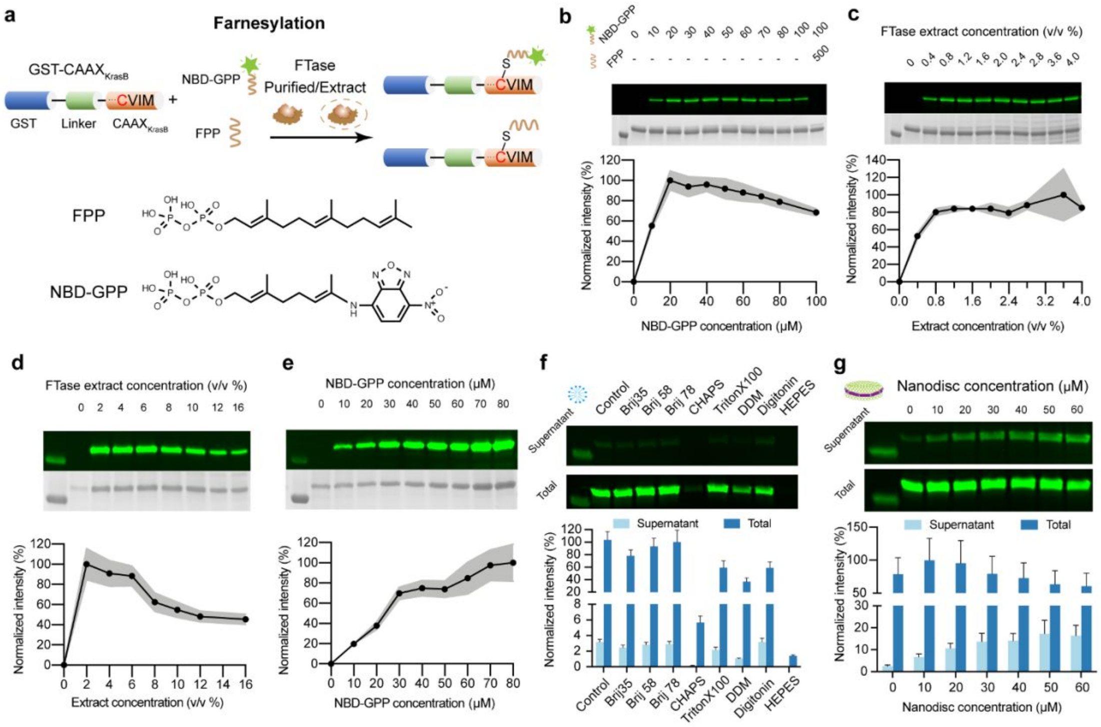

Figure S4 Establishment of cell-free prenylation protein synthesis (CFpPS) system for farnesylation. a) Schematic illustration of the chimeric proteins GST-CAAX$_{KrasB}$ that are farnesylated via purified FTase or FTase-enriched extracts. **b)** Titration of the NBD-GPP with purified FTase using in-gel fluorescence. Last lane showed the competition assay performed by adding the unlabeled analogue—FPP—at a 5-fold concentration of the highest tested for NBD-modified analogue. Concentration (μM) of lipid donor in each reaction is stated above the corresponding gel lane. **c)** Titration of FTase-enriched extracts using In-gel fluorescence with 10 μM GST-CAAX$_{KrasB}$ and 20 μM NBD-GPP. Extract concentration is shown as percentage volume of FTase-enriched extract included in the standard *E. coli* CFPS. **d)** Concentration optimization of FTase-enriched extract in CFpPS system using in-gel fluorescence. Note that GST-CAAX$_{KrasB}$ is co-translationally prenylated in the CFpPS system. Extract concentration is shown as percentage volume of the enriched extract included in the standard E. coli CFPS. **e)** In-gel fluorescence analysis for optimizing the concentration of NBD-GPP in the CFpPS system. **f)** Screening of detergents for soluble expression of farnesylated GST-CAAX$_{KrasB}$. Respective control reactions were performed without any detergent. All intensity values were normalized using the highest averaged values. **g)** Nanodisc titration for the soluble expression of GST-CAAX$_{KrasB}$ in CFpPS system. Fluorescence intensities of the protein band for each fraction were measured through in-gel fluorescence and normalized to the highest averaged value. Each image panel (b-e) includes a representative gel imaged in fluorescence mode to visualize NBD (upper) and colorimetric mode to visualize Coomassie staining (lower). In all graphs (b-e), mean values from three independent replicates are shown as black dots, while the grey shading represents standard deviation, n=3.

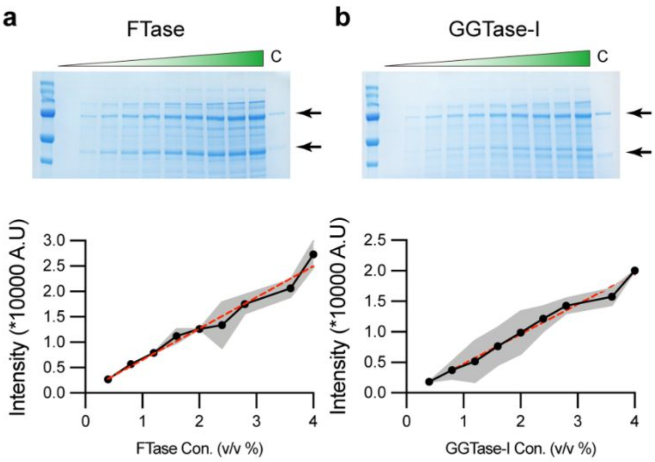

Figure S5. Crude estimation of the prenyltransferase concentration in enriched extracts. Coomassie-stained SDS-PAGE gels (upper row) of the serial dilution of either Ftase- **a)** or GGTase-I-enriched **b)** extract. A control sample (lane C) corresponding to the respective purified prenyltransferase and with a known concentration is shown for comparison (0.4 uM FTase, 2 uM GGTase-I). Lower panel: Estimation of the prenylatransfease concentration in the enriched extracts through colorimetry density estimation. Black arrows indicate the bands corresponding to the α and β subunit of the prenyltransferases.

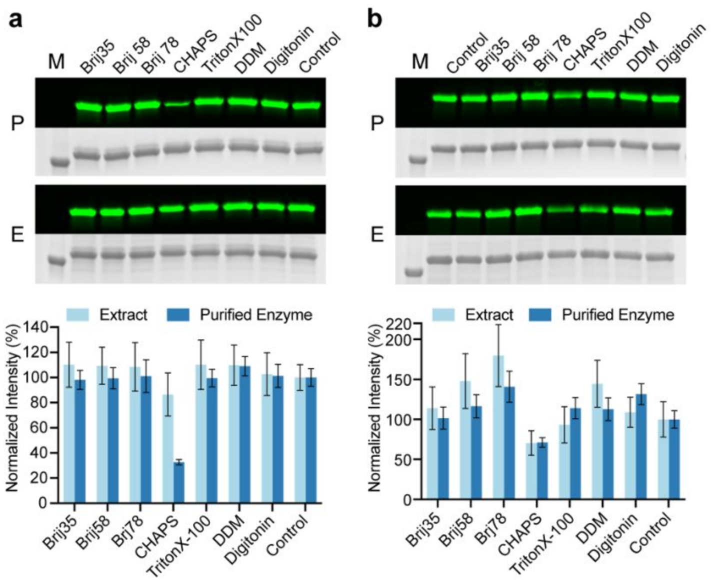

Figure S6. Detergent compatibility for *in vitro* prenylation reactions. Detergent compatibility for farnesylation **a)** and for geranylgeranylation **b)**. P denotes purified prenyltransferase, E denotes prenyltransferase-enriched extract and M denotes protein marker. In-gel fluorescence of NBD-labeled isoprenoid group was visualized for each modified protein band. The intensity of each fluorescent band was calibrated by densitometry from the corresponding Coomassie-stained gel images and normalized according to the Control without any detergent. Bar graphs display mean values and standard deviations are shown, n = 3 independent replicates.

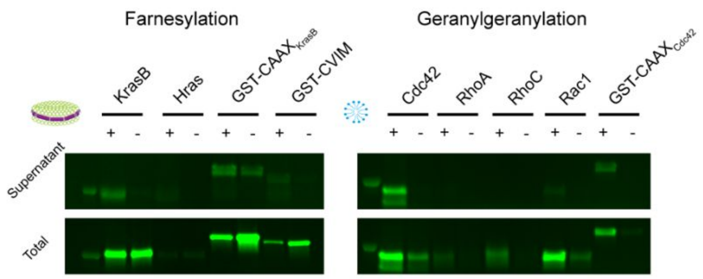

Figure S7. In-gel fluorescence analysis of the expression and solubilization of representative proteins. NBD-labeled isoprenoid group was used as lipid donor and visualized by in-gel fluorescence. Proteins correspond to those listed in Table S1. Either nanodiscs (left panel) or the detergent Brij58 (right panel) was used for farnesylation and geranylgeranylation of the representative CAAX proteins, respectively.

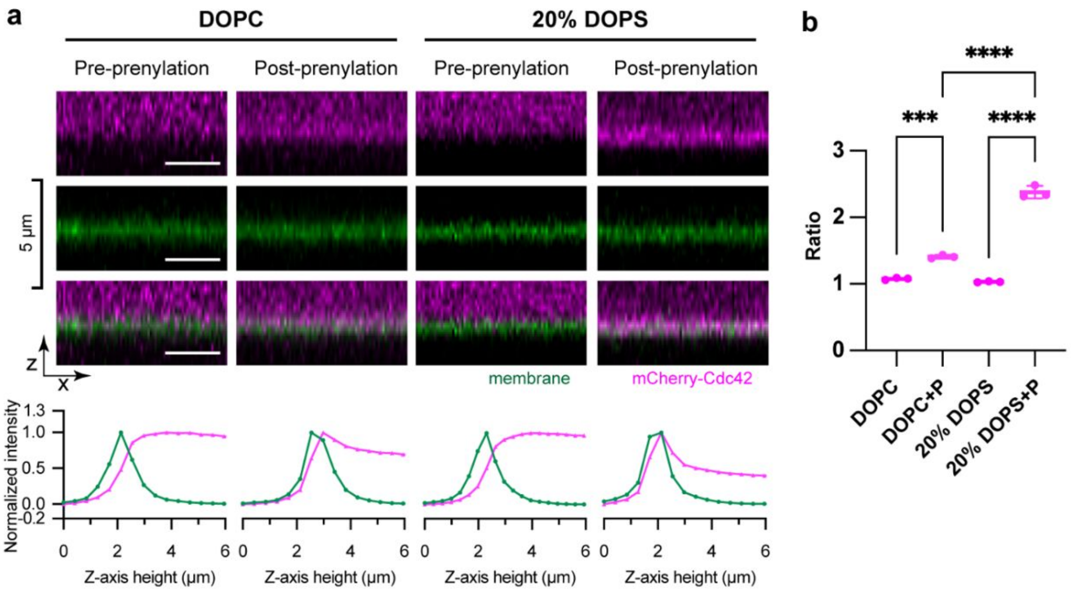

Figure S8. Charge-dependent targeting of mCherry-Cdc42 to supported lipid bilayers before and after prenylation. a) Orthogonal views (upper panel) of mCherry-Cdc42 binding to neutral (100 % DOPC) or negatively charged (20% DOPS, 80% DOPC) supported lipid bilayers (SLB) before and after the addition of the lipid donor GGPP, membrane channel was visualized by the addition of 0.05 mol% ATTO488-DOPE. Lower panel: Normalized intensities of the respective Z-stacks for each experimental condition. **b)** Ratio of the mCherry-Cdc42 intensity averaged over the Z-slice corresponding to the membrane and averaged over the slice of the stack furthest from the membrane (in solution). Abbreviations DOPC and 20 % DOPS stand for supported lipid bilayers containing only neural lipids or 20 % of negatively charged ones, respectively. "+P" indicates the presence of the lipid donor GGPP and hence a prenylated protein state. Statistical analysis of the determined ratios was performed by a one-way ANOVA followed by multiple comparisons. P values of 0.0001 are highlighted by *** while **** indicate P values of <0.0001, n = 3 independent replicates.

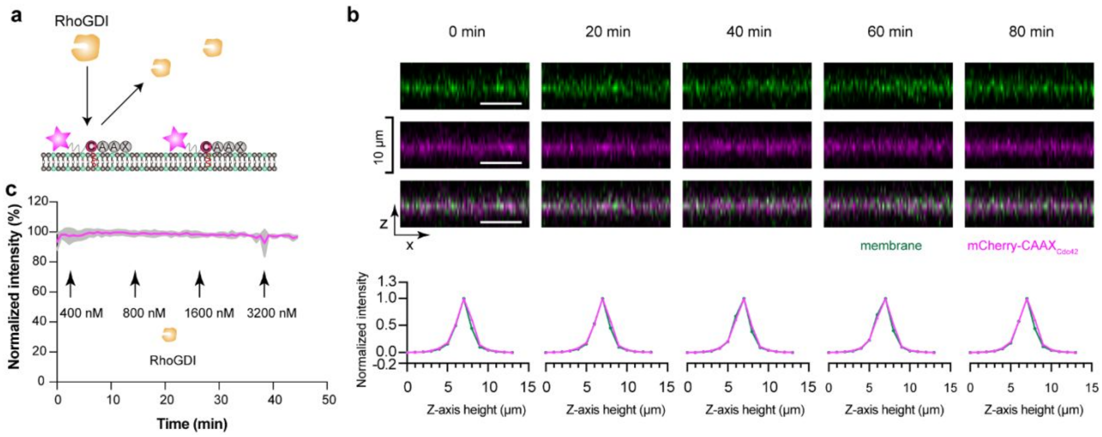

Figure S9. RhoGDI fails to extract mCherry-CAAX$_{Cdc42}$ from the membrane. a) Schematic illustration showing that unlike full-length Cdc42 (Fig. 4), RhoGDI cannot bind to mCherry-CAAX$_{Cdc42}$ and extract it from the membrane. mCherry-CAAX$_{Cdc42}$ lacks the interface required for RhoGDI interaction of Cdc42. **b)** Orthogonal view of representative fluorescent images (upper panel) of membrane-localized mCherry-CAAX$_{Cdc42}$ in the presence of RhoGDI. Lower panel, normalized fluorescence intensities of the corresponding images from upper panel. Images correspond to time since the start of imaging of an SLB (80% DOPC, 19.95% DOPS, 0.05% Atto-488 PE) loaded with prenylated mCherry-CAAX$_{Cdc42.}$ RhoGDI was added in increments at times observed in panel **c**. The image panels show the membrane in green (upper row), mCherry-tagged protein in magenta (middle row) and a merge of both channels (lower row). Scale bar: 10 µm. **c)** Normalized average intensity of the mCherry-CAAX$_{Cdc42}$ signal in the presence of RhoGDI. Standard deviations are indicated in gray shades (n=3) and a representative dataset of 3 independent replicates is shown. Intensity values were obtained by averaging over the slice that corresponds to the membrane and then normalized by setting the maximum and minimum intensities recorded during each experiment as 0 and 1, respectively. The indicated RhoGDI concentrations represent freshly added protein into the chamber at each time point besides the pre-existing RhoGDI.

Supplementary Tables

Table S1 List of the protein constructs used in this study

Protein name	Vector	Source
FTase α	pGEXTEV-FTase-α	Dursina, et al.[1]
FTase β	pET28-FTase-β	Dursina, et al.[1]
GGTase-I α	pGEXTEV-GGTase-I-α	Dursina, et al.[1]
GGTase-I β	pET28-GGTase-I-β	Dursina, et al.[1]
GST-CAAX$_{KrasB}$	pGEX-RG-KrasB-C1-15	This study
GST-CAAX$_{Cdc42}$	pGEX-RG-hCdc42-C1-9	This study
RhoGDI	pCoofy1_Linker-hRhoGDI	This study
mCherry-CAAX$_{Cdc42}$	pCoofy1a-mCherry-RG-Caax-CVLL	This study
RhoA	pIVEX2.3d-RhoA	This study
RhoC	pIVEX2.3d-RhoC	This study
Rac1	pIVEX2.3d-Rac1	This study
KrasB	pIVEX2.3d-KrasB	This study
HRas	pIVEX2.3d-HRas	This study
Cdc42	pIVEX2.3d-Cdc42	This study
mCherry-Cdc42	pCoofy1a-mCherry-Cdc42	This study
MSP1E3D1	pET28a-MSP1E3D1	Li, et al.[2]

Table S2. Summary of the CFpPS system's performance in expressing and solubilizing chimeric CAAX-proteins and native small GTPases.

Constructs	C-terminal sequences	Type of Prenylation	Detected Modification	Soluble Modification
GST-CAAX$_{KrasB}$	GKKKKKKSKTKCVIM	F/G	+	+
GST-CVIM	GCVIM	F/G	+	+
GST-CAAX$_{Cdc42}$	KKSRRCVLL	G	+	+
KrasB	GKKKKKKSKTKCVIM	F/G	+	+
Hras	CVLS	F	+	+
Cdc42	KKSRRCVLL	G	+	+
RhoA	RRGKKKSGCLVL	G	+	-
RhoC	KNKRRRGCPIL	G	+	-
Rac1	KKRKRKCLLL	G	+	+

F, farnesylation; G, geranylgeranylation.

Gene and protein sequences

Record of the complete sequences of genes and proteins that have been used or created in this study. For fusion constructs, linker sequences are shown in italics. The 3C protease recognition sequences are highlighted with underline. C-terminal sequences of KrasB and Cdc42 are shown in bold in chimeric constructs.

FTase α

Protein sequence

MSPILGYWKIKGLVQPTRLLLEYLEEKYEEHLYERDEGDKWRNKKFELGLEFPNLPYYID
GDVKLTQSMAIIRYIADKHNMLGGCPKERAEISMLEGAVLDIRYGVSRIAYSKDFETLKV
DFLNKLPEMLKMFEDRLCHKTYLNGDHVTHPDFMLYDALDVVLYMDPMCLDAFPKLV
CFKKRIEAIPQIDKYLKSSKYIAWPLQGWQATFGGGDHPPTNRWVSMADENLYFQGHMA
ATEGVGESAPGGEPGQPEQPPPPPPPPPAQQPQEEEMAAEAGEAAASPMDDGFLSLDSPT
YVLYRDRAEWADIDPVPQNDGPSPVVQIIYSEKFRDVYDYFRAVLQRDERSERAFKLTRD
AIELNAANYTVWHFRRVLLRSLQKDLQEEMNYITAIIEEQPKNYQVWHHRRVLVEWLKD
PSQELEFIADILNQDAKNYHAWQHRQWVIQEFRLWDNELQYVDQLLKEDVRNNSVWNQ
RHFVISNTTGYSDRAVLEREVQYTLEMIKLVPHNESAWNYLKGILQDRGLSRYPNLLNQL
LDLQPSHSSPYLIAFLVDIYEDMLENQCDNKEDILNKALELCEILAKEKDTIRKEYWRYIG
RSLQSKHSRESDIPASV*

DNA sequence

ATGTCCCCTATACTAGGTTATTGGAAAATTAAGGGCCTTGTGCAACCCACTCGACTTC
TTTTGGAATATCTTGAAGAAAAATATGAAGAGCATTTGTATGAGCGCGATGAAGGTG
ATAAATGGCGAAACAAAAAGTTTGAATTGGGTTTGGAGTTTCCCAATCTTCCTTATTA
TATTGATGGTGATGTTAAATTAACACAGTCTATGGCCATCATACGTTATATAGCTGAC
AAGCACAACATGTTGGGTGGTTGTCCAAAAGAGCGTGCAGAGATTTCAATGCTTGAA
GGAGCGGTTTTGGATATTAGATACGGTGTTTCGAGAATTGCATATAGTAAAGACTTTG
AAACTCTCAAAGTTGATTTTCTTAACAAGCTACCTGAAATGCTGAAAATGTTCGAAGA
TCGTTTATGTCATAAAACATATTTAAATGGTGATCATGTAACCCATCCTGACTTCATG
TTGTATGACGCTCTTGATGTTGTTTTATACATGGATCCAATGTGCCTGGATGCGTTCCC
AAAATTAGTTTGTTTTAAAAAACGTATTGAAGCTATCCCACAAATTGATAAGTACTTG
AAATCCAGCAAGTATATAGCATGGCCTTTGCAGGGCTGGCAAGCCACGTTTGGTGGT
GGCGACCATCCTCCAACTAATCGATGGGTATCCATGGCTGATGAGAATCTTTATTTTC
AGGGCCATATGGCGGCCACTGAGGGGGTCGGGGAATCTGCGCCAGGCGGTGAGCCG
GGACAGCCAGAGCAGCCGCCGCCCCCGCCTCCTCCGCCGCCAGCACAGCAGCCGCAG
GAAGAAGAGATGGCGGCCGAGGCCGGGGAAGCAGCGGCGTCCCCTATGGACGACGG
GTTTCTGAGCCTGGACTCGCCCACCTATGTCTTGTACAGGGACAGGGCAGAGTGGGCT
GACATAGACCCAGTGCCCCAGAATGATGGCCCCAGTCCAGTGGTCCAGATCATCTAC
AGTGAAAAGTTTAGAGACGTCTATGATTACTTCCGAGCTGTTCTGCAGCGCGATGAA
AGAAGCGAACGAGCCTTTAAGCTCACTCGAGATGCTATTGAGTTAAACGCAGCCAAC
TATACGGTGTGGCATTTTCGGAGAGTTCTCTTGAGGTCGCTTCAGAAGGATCTGCAAG
AAGAAATGAACTACATCACTGCAATAATTGAGGAACAGCCCAAAAACTATCAAGTTT
GGCACCATAGGAGAGTATTAGTGGAGTGGCTGAAAGATCCTTCTCAAGAGCTCGAGT
TCATCGCCGATATCCTTAATCAGGATGCAAAGAATTACCATGCCTGGCAGCATCGAC
AGTGGGTCATTCAGGAGTTTCGACTTTGGGATAATGAGCTGCAGTATGTGGACCAGCT

TCTCAAAGAGGATGTGAGAAATAACTCTGTGTGGAACCAAAGACACTTCGTCATTTCT
AATACCACTGGCTACAGTGATCGCGCTGTGTTGGAGAGAGAAGTCCAATATACTCTG
GAAATGATCAAATTAGTGCCACACAATGAGAGTGCGTGGAACTACTTGAAAGGGATT
TTGCAGGACCGTGGTCTTTCCAGATACCCTAATCTATTAAACCAGTTGCTTGATTTAC
AACCAAGTCACAGCTCCCCCTACCTAATTGCCTTTCTTGTGGATATCTATGAAGACAT
GCTGGAAAACCAGTGTGACAACAAGGAGGACATTCTTAATAAAGCACTAGAGTTATG
TGAGATTCTAGCTAAAGAAAAGGACACTATAAGAAAGGAATATTGGAGATATATTGG
ACGGTCCCTCCAGAGTAAACACAGCAGAGAAAGTGACATACCGGCGAGTGTATAG

FTase β

Protein sequence

MASSSSFTYYCPPSSSPVWSEPLYSLRPEHARERLQDDSVETVTSIEQAKVEEKIQEVFSSY
KFNHLVPRLVLQREKHFHYLKRGLRQLTDAYECLDASRPWLCYWILHSLELLDEPIPQIV
ATDVCQFLELCQSPDGGFGGGPGQYPHLAPTYAAVNALCIIGTEEAYNVINREKLLQYLY
SLKQPDGSFLMHVGGEVDVRSAYCAASVASLTNIITPDLFEGTAEWIARCQNWEGGIGGV
PGMEAHGGYTFCGLAALVILKKERSLNLKSLLQWVTSRQMRFEGGFQGRCNKLVDGCY
SFWQAGLLPLLHRALHAQGDPALSMSHWMFHQQALQEYILMCCQCPAGGLLDKPGKSR
DFYHTCYCLSGLSIAQHFGSGAMLHDVVMGVPENVLQPTHPVYNIGPDKVIQATTHFLQ
KPVPGFEECEDAVTSDPATD*

DNA sequence

ATGGCTTCTTCGAGTTCCTTCACCTATTATTGTCCTCCATCTTCTTCCCCTGTTTGGTCA
GAACCGCTGTATAGTCTGAGACCTGAGCACGCGCGGGAGCGGTTGCAAGACGACTCA
GTGGAAACAGTCACGTCCATAGAACAGGCCAAAGTAGAAGAAAAGATCCAGGAGGT
CTTCAGTTCTTACAAGTTTAACCACCTCGTACCAAGGCTCGTTCTGCAGAGGGAGAAG
CACTTCCATTATCTGAAAAGAGGCCTTCGACAACTGACAGATGCCTATGAGTGTCTGG
ATGCCAGCCGCCCCTGGCTCTGCTACTGGATCCTGCACAGCTTGGAGCTCCTCGACGA
ACCCATCCCCCAAATAGTGGCTACAGATGTGTGTCAGTTCTTGGAGCTGTGTCAGAGT
CCAGACGGTGGCTTTGGAGGGGGCCCTGGTCAGTACCCACACCTCGCTCCCACGTAT
GCAGCTGTCAACGCGCTATGCATCATTGGCACGGAGGAAGCCTACAACGTCATTAAC
AGAGAGAAGCTCCTTCAGTACTTGTACTCCCTAAAGCAACCGGATGGCTCTTTTCTCA
TGCACGTCGGAGGAGAGGTGGATGTAAGAAGTGCGTACTGTGCTGCCTCAGTAGCCT
CTCTCACCAACATCATCACTCCTGACCTCTTCGAAGGCACTGCTGAATGGATAGCAAG
GTGCCAGAACTGGGAAGGCGGCATTGGCGGGGTGCCAGGGATGGAAGCCCACGGTG
GCTACACCTTCTGTGGCTTGGCTGCGCTGGTGATCCTCAAGAAGGAACGTTCTTTGAA
CCTGAAGAGCTTGCTACAATGGGTGACAAGCCGGCAGATGCGGTTCGAAGGAGGATT
TCAGGGCCGCTGCAACAAGCTGGTGGACGGCTGCTACTCCTTCTGGCAGGCAGGACT
TCTGCCCCTGTTGCACCGGGCACTCCACGCTCAAGGTGACCCTGCCCTCAGCATGAGC
CACTGGATGTTCCATCAGCAGGCGCTGCAGGAGTACATCCTCATGTGCTGCCAGTGTC
CGGCTGGGGGTCTCCTGGACAAACCTGGCAAGTCACGTGACTTCTACCATACTTGCTA
CTGCCTGAGCGGCCTGTCCATTGCCCAGCATTTTGGAAGTGGAGCCATGCTGCACGAT
GTGGTCATGGGTGTGCCTGAAAATGTTCTGCAGCCCACTCACCCTGTGTACAACATCG
GACCTGATAAGGTGATCCAGGCCACCACACACTTTCTGCAGAAGCCGGTCCCAGGCT
TTGAGGAATGCGAAGATGCGGTGACCTCAGATCCTGCCACTGACTAG

GGTase-I beta

Protein sequence
MAATEDDRLAGSGEGERLDFLRDRHVRFFQRCLQVLPERYSSLETSRLTIAFFALSGLDM
LDSLDVVNKDDIIEWIYSLQVLPTEDRSNLDRCGFRGSSYLGIPFNPSKNPGTAHPYDSGHI
AMTYTGLSCLIILGDDLSRVDKEACLAGLRALQLEDGSFCAVPEGSENDMRFVYCASCIC
YMLNNWSGMDMKKAISYIRRSMSYDNGLAQGAGLESHGGSTFCGIASLCLMGKLEEVFS
EKELNRIKRWCIMRQQNGYHGRPNKPVDTCYSFWVGATLKLLKIFQYTNFEKNRNYILST
QDRLVGGFAKWPDSHPDALHAYFGICGLSLMEESGICKVHPALNVSTRTSERLRDLHQS
WKTKDSKQCSDNVHISS*
DNA sequence
ATGGCGGCCACAGAGGATGACAGACTGGCGGGGAGCGGAGAAGGAGAACGGCTGGA
TTTCCTGCGGGACCGACACGTGCGGTTCTTCCAGCGCTGCCTCCAGGTCTTGCCGGAG
CGGTATTCTTCGCTGGAGACCAGCAGGCTGACAATTGCATTTTTGCACTCTCTGGGC
TGGATATGTTGGACTCCTTGGATGTGGTGAACAAAGACGATATAATAGAGTGGATTT
ATTCCTTGCAGGTTCTTCCCACAGAAGACAGGTCAAATCTGGATCGCTGTGGTTTCCG
AGGTTCTTCATATTTGGGTATTCCATTCAACCCATCAAAGAATCCAGGCACAGCTCAT
CCTTATGACAGTGGACACATAGCGATGACTTACACTGGTCTTTCCTGTTTAATTATTCT
TGGAGATGATTTAAGCCGAGTAGATAAAGAAGCTTGCTTAGCAGGCTTGAGAGCACT
TCAGCTGGAAGATGGGAGCTTCTGTGCTGTTCCTGAAGGCAGTGAGAATGACATGAG
GTTTGTGTACTGTGCTTCCTGCATTTGCTATATGCTCAACAACTGGTCAGGCATGGAT
ATGAAGAAAGCCATCAGCTACATTAGAAGAAGTATGTCCTATGACAATGGCCTGGCA
CAGGGGGCAGGACTTGAGTCTCATGGAGGATCCACCTTTTGTGGCATTGCGTCACTGT
GCCTGATGGGTAAACTGGAAGAAGTTTTTCAGAGAAAGAACTGAACCGGATAAAGA
GGTGGTGCATAATGAGGCAGCAGAACGGGTACCACGGAAGACCTAACAAGCCTGTC
GACACCTGTTACTCTTTCTGGGTGGGAGCAACACTAAAGCTTTTGAAAATTTTCCAGT
ACACTAACTTTGAGAAGAATAGGAATTACATCTTATCAACTCAGGATCGCCTTGTTGG
GGGATTTGCTAAATGGCCAGACAGTCATCCAGATGCTTTGCATGCGTACTTCGGGATC
TGTGGCCTGTCACTAATGGAGGAGAGTGGAATTTGTAAAGTTCATCCTGCTCTGAATG
TAAGCACACGAACTTCTGAGCGCCTCCGAGATCTCCATCAAAGCTGGAAGACCAAGG
ACTCTAAACAGTGCTCAGACAATGTCCATATTTCCAGTTGA

GST-CAAX$_{\mathbf{KrasB}}$
Protein sequence
MNTIHHHHHHNTSSNSMSPILGYWKIKGLVQPTRLLLEYLEEKYEEHLYERDEGDKWRN
KKFELGLEFPNLPYYIDGDVKLTQSMAIIRYIADKHNMLGGCPKERAEISMLEGAVLDIRY
GVSRIAYSKDFETLKVDFLNKLPEMLKMFEDRLCHKTYLNGDHVTHPDFMLYDALDVV
LYMDPMCLDAFPKLVCFKKRIEAIPQIDKYLKSSKYIAWPLQGWQATFGGGDHPPGSAG*L*
*AEAAAKEAAAKEAAAKEAAAKAAA***GKKKKKKSKTKCVIM***
DNA sequence
ATGAACACCATTCATCACCATCACCATCACAACACTAGTAGCAATTCCATGTCCCCTA
TACTAGGTTATTGGAAAATTAAGGGCCTTGTGCAACCCACTCGACTTCTTTTGGAATA
TCTTGAAGAAAAATATGAAGAGCATTTGTATGAGCGCGATGAAGGTGATAAATGGCG
AAACAAAAAGTTTGAATTGGGTTTGGAGTTTCCCAATCTTCCTTATTATATTGATGGT
GATGTTAAATTAACACAGTCTATGGCCATCATACGTTATATAGCTGACAAGCACAAC
ATGTTGGGTGGTTGTCCAAAAGAGCGTGCAGAGATTTCAATGCTTGAAGGAGCGGTT

TTGGATATTAGATACGGTGTTTCGAGAATTGCATATAGTAAAGACTTTGAAACTCTCA
AAGTTGATTTTCTTAACAAGCTACCTGAAATGCTGAAAATGTTCGAAGATCGTTTATG
TCATAAAACATATTTAAATGGTGATCATGTAACCCATCCTGACTTCATGTTGTATGAC
GCTCTTGATGTTGTTTTATACATGGATCCAATGTGCCTGGATGCGTTCCCAAAATTAG
TTTGTTTTAAAAAACGTATTGAAGCTATCCCACAAATTGATAAGTACTTGAAATCCAG
CAAGTATATAGCATGGCCTTTGCAGGGCTGGCAAGCCACGTTTGGTGGTGGCGACCA
TCCTCCAGGTTCTGCGGGTTTAGCAGAGGCGGCAGCTAAAGAAGCTGCTGCCAAAGA
AGCTGCCGCGAAAGAAGCGGCTGCCAAGGCTGCGGCAGGCAAGAAAAAGAAAAAGA
AAAGCAAGACAAAATGTGTAATCATGTGA

GST-CAAX$_{Cdc42}$

Protein sequence

MNTIHHHHHHNTSSNSMSPILGYWKIKGLVQPTRLLLEYLEEKYEEHLYERDEGDKWRN
KKFELGLEFPNLPYYIDGDVKLTQSMAIIRYIADKHNMLGGCPKERAEISMLEGAVLDIRY
GVSRIAYSKDFETLKVDFLNKLPEMLKMFEDRLCHKTYLNGDHVTHPDFMLYDALDVV
LYMDPMCLDAFPKLVCFKKRIEAIPQIDKYLKSSKYIAWPLQGWQATFGGGDHPPGSAG*L*
*AEAAAKEAAAKEAAAKEAAAKAAA***KKSRRCVLL***

DNA sequence

ATGAACACCATTCATCACCATCACCATCACAACACTAGTAGCAATTCCATGTCCCCTA
TACTAGGTTATTGGAAAATTAAGGGCCTTGTGCAACCCACTCGACTTCTTTTGGAATA
TCTTGAAGAAAAATATGAAGAGCATTTGTATGAGCGCGATGAAGGTGATAAATGGCG
AAACAAAAAGTTTGAATTGGGTTTGGAGTTTCCCAATCTTCCTTATTATATTGATGGT
GATGTTAAATTAACACAGTCTATGGCCATCATACGTTATATAGCTGACAAGCACAAC
ATGTTGGGTGGTTGTCCAAAAGAGCGTGCAGAGATTTCAATGCTTGAAGGAGCGGTT
TTGGATATTAGATACGGTGTTTCGAGAATTGCATATAGTAAAGACTTTGAAACTCTCA
AAGTTGATTTTCTTAACAAGCTACCTGAAATGCTGAAAATGTTCGAAGATCGTTTATG
TCATAAAACATATTTAAATGGTGATCATGTAACCCATCCTGACTTCATGTTGTATGAC
GCTCTTGATGTTGTTTTATACATGGATCCAATGTGCCTGGATGCGTTCCCAAAATTAG
TTTGTTTTAAAAAACGTATTGAAGCTATCCCACAAATTGATAAGTACTTGAAATCCAG
CAAGTATATAGCATGGCCTTTGCAGGGCTGGCAAGCCACGTTTGGTGGTGGCGACCA
TCCTCCAGGTTCTGCGGGTTTAGCAGAGGCGGCAGCTAAAGAAGCTGCTGCCAAAGA
AGCTGCCGCGAAAGAAGCGGCTGCCAAGGCTGCGGCAAAGAAATCCAGGCGGTGCG
TTCTGCTGTGA

GST-CVIM

Protein sequence

MNTIHHHHHHNTSSNSMSPILGYWKIKGLVQPTRLLLEYLEEKYEEHLYERDEGDKWRN
KKFELGLEFPNLPYYIDGDVKLTQSMAIIRYIADKHNMLGGCPKERAEISMLEGAVLDIRY
GVSRIAYSKDFETLKVDFLNKLPEMLKMFEDRLCHKTYLNGDHVTHPDFMLYDALDVV
LYMDPMCLDAFPKLVCFKKRIEAIPQIDKYLKSSKYIAWPLQGWQATFGGGDHPPGSAG*L*
*AEAAAKEAAAKEAAAKEAAAKAAA***CVIM***

DNA sequence

ATGTCCCCTATACTAGGTTATTGGAAAATTAAGGGCCTTGTGCAACCCACTCGACTTC
TTTTGGAATATCTTGAAGAAAAATATGAAGAGCATTTGTATGAGCGCGATGAAGGTG

ATAAATGGCGAAACAAAAAGTTTGAATTGGGTTTGGAGTTTCCCAATCTTCCTTATTA
TATTGATGGTGATGTTAAATTAACACAGTCTATGGCCATCATACGTTATATAGCTGAC
AAGCACAACATGTTGGGTGGTTGTCCAAAAGAGCGTGCAGAGATTTCAATGCTTGAA
GGAGCGGTTTTGGATATTAGATACGGTGTTTCGAGAATTGCATATAGTAAAGACTTTG
AAACTCTCAAAGTTGATTTTCTTAACAAGCTACCTGAAATGCTGAAAATGTTCGAAGA
TCGTTTATGTCATAAAACATATTTAAATGGTGATCATGTAACCCATCCTGACTTCATG
TTGTATGACGCTCTTGATGTTGTTTTATACATGGATCCAATGTGCCTGGATGCGTTCCC
AAAATTAGTTTGTTTTAAAAAACGTATTGAAGCTATCCCACAAATTGATAAGTACTTG
AAATCCAGCAAGTATATAGCATGGCCTTTGCAGGGCTGGCAAGCCACGTTTGGTGGT
GGCGACCATCCTCCAGGTTCTGCGGGTTTAGCAGAGGCGGCAGCTAAAGAAGCTGCT
GCCAAAGAAGCTGCCGCGAAAGAAGCGGCTGCCAAGGCTGCGGCATGTGTAATCATG
TGA

RhoGDI

Protein sequence

MSPILGYWKIKGLVQPTRLLLEYLEEKYEEHLYERDEGDKWRNKKFELGLEFPNLPYYID
GDVKLTQSMAIIRYIADKHNMLGGCPKERAEISMLEGAVLDIRYGVSRIAYSKDFETLKV
DFLSKLPEMLKMFEDRLCHKTYLNGDHVTHPDFMLYDALDVVLYMDPMCLDAFPKLVC
FKKRIEAIPQIDKYLKSSKYIAWPLQGWQATFGGGDHPPKSDLEVLFQGPLGSPEFMAEQE
PTAEQLAQIAAENEEDEHSVNYKPPAQKSIQEIQELDKDDESLRKYKEALLGRVAVSADP
NVPNVVVTGLTLVCSSAPGPLELDLTGDLESFKKQSFVLKEGVEYRIKISFRVNREIVSGM
KYIQHTYRKGVKIDKTDYMVGSYGPRAEEYEFLTPVEEAPKGMLARGSYSIKSRFTDDD
KTDHLSWEWNLTIKKDWKD*

DNA sequence

ATGTCCCCTATACTAGGTTATTGGAAAATTAAGGGCCTTGTGCAACCCACTCGACTTC
TTTTGGAATATCTTGAAGAAAAATATGAAGAGCATTTGTATGAGCGCGATGAAGGTG
ATAAATGGCGAAACAAAAAGTTTGAATTGGGTTTGGAGTTTCCCAATCTTCCTTATTA
TATTGATGGTGATGTTAAATTAACACAGTCTATGGCCATCATACGTTATATAGCTGAC
AAGCACAACATGTTGGGTGGTTGTCCAAAAGAGCGTGCAGAGATTTCAATGCTTGAA
GGAGCGGTTTTGGATATTAGATACGGTGTTTCGAGAATTGCATATAGTAAAGACTTTG
AAACTCTCAAAGTTGATTTTCTTAGCAAGCTACCTGAAATGCTGAAAATGTTCGAAGA
TCGTTTATGTCATAAAACATATTTAAATGGTGATCATGTAACCCATCCTGACTTCATG
TTGTATGACGCTCTTGATGTTGTTTTATACATGGACCCAATGTGCCTGGATGCGTTCCC
AAAATTAGTTTGTTTTAAAAAACGTATTGAAGCTATCCCACAAATTGATAAGTACTTG
AAATCCAGCAAGTATATAGCATGGCCTTTGCAGGGCTGGCAAGCCACGTTTGGTGGT
GGCGACCATCCTCCAAAATCGGATCTGGAAGTTCTGTTCCAGGGGCCCCTGGGATCCC
CGGAATTCATGGCCGAGCAAGAACCGACTGCAGAACAACTTGCGCAAATTGCAGCGG
AAAACGAGGAAGATGAGCATAGCGTGAACTACAAACCACCAGCCCAGAAAAGCATT
CAGGAAATTCAGGAGCTGGATAAAGACGATGAATCGCTGCGGAAATACAAAGAAGC
CCTCTTAGGTCGTGTAGCGGTTTCAGCGGATCCGAATGTCCCGAATGTTGTGGTGACT
GGCCTGACGTTGGTCTGCAGCAGTGCTCCTGGTCCGTTAGAGCTGGATCTGACGGGTG
ATCTGGAATCGTTCAAGAAACAGAGCTTTGTCCTGAAAGAAGGGGTGGAATATCGCA
TCAAAATCTCTTTTCGCGTAAATCGCGAAATTGTGTCTGGCATGAAATACATTCAGCA
CACCTATCGCAAAGGCGTGAAAATCGACAAAACGGACTATATGGTTGGATCGTATGG

TCCTCGTGCGGAAGAGTATGAGTTCCTCACACCGGTTGAAGAAGCACCCAAAGGCAT
GCTTGCTCGTGGGTCCTACTCCATTAAGTCACGCTTTACCGACGATGACAAGACCGAT
CATCTGAGTTGGGAATGGAACTTGACCATCAAGAAAGACTGGAAAGATTGA

mCherry-CAAX$_{Cdc42}$

Protein sequence

MKHHHHHHHHHHSAGLEVLFQGPMVSKGEEDNMAIIKEFMRFKVHMEGSVNGHEFEIE
GEGEGRPYEGTQTAKLKVTKGGPLPFAWDILSPQFMYGSKAYVKHPADIPDYLKLSFPEG
FKWERVMNFEDGGVVTVTQDSSLQDGEFIYKVKLRGTNFPSDGPVMQKKTMGWEASSE
RMYPEDGALKGEIKQRLKLKDGGHYDAEVKTTYKAKKPVQLPGAYNVNIKLDITSHNED
YTIVEQYERAEGRHSTGGMDELYK*LAEAAAKEAAAKEAAAKEAAAKAAA*G**CVLL***

DNA sequence

ATGAAACATCACCATCACCATCACCATCATCACCATTCCGCGGGTCTGGAAGTTCTGT
TCCAGGGGCCCATGGTGAGCAAGGGCGAGGAGGATAACATGGCCATCATCAAGGAG
TTCATGCGCTTCAAGGTGCACATGGAGGGCTCCGTGAACGGCCACGAGTTCGAGATC
GAGGGCGAGGGCGAGGGCCGCCCCTACGAGGGCACCCAGACCGCCAAGCTGAAGGT
GACCAAGGGTGGCCCCCTGCCCTTCGCCTGGGACATCCTGTCCCCTCAGTTCATGTAC
GGCTCCAAGGCCTACGTGAAGCACCCCGCCGACATCCCCGACTACTTGAAGCTGTCCT
TCCCCGAGGGCTTCAAGTGGGAGCGCGTGATGAACTTCGAGGACGGCGGCGTGGTGA
CCGTGACCCAGGACTCCTCCCTGCAGGACGGCGAGTTCATCTACAAGGTGAAGCTGC
GCGGCACCAACTTCCCCTCCGACGGCCCCGTAATGCAGAAGAAGACCATGGGCTGGG
AGGCCTCCTCCGAGCGGATGTACCCCGAGGACGGCGCCCTGAAGGGCGAGATCAAGC
AGAGGCTGAAGCTGAAGGACGGCGGCCACTACGACGCTGAGGTCAAGACCACCTAC
AAGGCCAAGAAGCCCGTGCAGCTGCCCGGCGCCTACAACGTCAACATCAAGTTGGAC
ATCACCTCCCACAACGAGGACTACACCATCGTGGAACAGTACGAACGCGCCGAGGGC
CGCCACTCCACCGGCGGCATGGACGAGCTGTACAAGTTAGCAGAGGCGGCAGCTAAA
GAAGCTGCTGCCAAAGAAGCTGCCGCGAAAGAAGCGGCTGCCAAGGCTGCGGCAGG
GTGCGTTCTGCTTTGA

RhoA

Protein sequence

MKHHHHHHHHHHSAGLEVLFQGPMAAIRKKLVIVGDGACGKTCLLIVFSKDQFPEVYVP
TVFENYVADIEVDGKQVELALWDTAGQEDYDRLRPLSYPDTDVILMCFSIDSPDSLENIPE
KWTPEVKHFCPNVPIILVGNKKDLRNDEHTRRELAKMKQEPVKPEEGRDMANRIGAFGY
MECSAKTKDGVREVFEMATRAALQARRGKKKSGCLVL*

DNA sequence

ATGAAACATCACCATCACCATCACCATCATCACCATTCCGCGGGTCTGGAAGTTCTGT
TCCAGGGGCCCATGGCGGCAATTCGCAAGAAACTGGTGATTGTAGGCGATGGTGCCT
GTGGCAAAACCTGTCTGCTGATCGTGTTCTCCAAAGACCAGTTTCCTGAAGTGTATGT
TCCCACCGTATTCGAAAACTACGTAGCGGACATTGAGGTTGATGGCAAACAGGTGGA
ATTAGCCCTGTGGGATACCGCAGGGCAAGAAGATTATGACCGGTTGCGCCCGTTAAG
CTATCCGGATACGGACGTCATCCTGATGTGCTTCAGCATCGATTCGCCAGATTCTCTC
GAGAACATTCCGGAGAAATGGACACCAGAAGTCAAACACTTTTGCCCGAATGTTCCG
ATTATCCTGGTGGGCAATAAGAAAGACCTTCGCAACGATGAGCATACCCGTCGCGAA

CTGGCGAAAATGAAACAGGAACCTGTCAAACCGGAAGAGGGACGTGACATGGCCAA
TCGCATTGGTGCGTTTGGGTACATGGAATGCAGTGCGAAAACGAAAGATGGTGTTCG
CGAAGTGTTTGAAATGGCCACTCGTGCTGCTCTCCAAGCACGTCGTGGTAAGAAGAA
ATCAGGCTGTTTGGTCCTGTGA

RhoC

Protein sequence

MKHHHHHHHHHHSAGLEVLFQGPMAAIRKKLVIVGDGACGKTCLLIVFSKDQFPEVYVP
TVFENYIADIEVDGKQVELALWDTAGQEDYDRLRPLSYPDTDVILMCFSIDSPDSLENIPE
KWTPEVKHFCPNVPIILVGNKKDLRQDEHTRRELAKMKQEPVRSEEGRDMANRISAFGY
LECSAKTKEGVREVFEMATRAGLQVRKNKRRRGCPIL*

DNA sequence

ATGAAACATCACCATCACCATCACCATCATCACCATTCCGCGGGTCTGGAAGTTCTGT
TCCAGGGGCCCATGGCAGCAATTCGCAAGAAACTGGTGATTGTCGGCGATGGTGCCT
GTGGCAAAACCTGTCTGCTGATTGTGTTCTCCAAAGACCAGTTTCCTGAAGTGTATGT
GCCGACAGTTTTCGAGAACTACATTGCGGATATCGAAGTAGACGGGAAACAGGTGGA
ACTGGCGTTATGGGATACGGCTGGTCAGGAAGATTACGATCGCCTGCGTCCGTTGAG
CTATCCGGATACCGATGTCATCCTCATGTGCTTCTCGATTGACAGCCCTGACAGTCTG
GAAAATATCCCCGAGAAATGGACGCCAGAAGTCAAGCACTTTTGCCCGAATGTACCG
ATCATCCTTGTAGGCAACAAGAAAGATCTGCGCCAAGACGAACATACCCGTCGCGAA
TTGGCGAAAATGAAACAAGAGCCAGTTCGGTCAGAAGAAGGTCGCGATATGGCTAAT
CGCATTTCGGCCTTTGGCTATCTGGAATGCTCTGCGAAAACCAAAGAAGGAGTTCGC
GAGGTGTTTGAGATGGCCACTCGTGCAGGGTTACAGGTTCGCAAGAACAAACGTCGT
CGGGGTTGTCCGATTCTCTAA

Rac1

Protein sequence

MKHHHHHHHHHHSAGLEVLFQGPMQAIKCVVVGDGAVGKTCLLISYTTNAFPGEYIPTV
FDNYSANVMVDGKPVNLGLWDTAGQEDYDRLRPLSYPQTDVFLICFSLVSPASFENVRA
KWYPEVRHHCPNTPIILVGTKLDLRDDKDTIEKLKEKKLTPITYPQGLAMAKEIGAVKYL
ECSALTQRGLKTVFDEAIRAVLCPPPVKKRKRKCLLL*

DNA sequence

ATGAAACATCACCATCACCATCACCATCATCACCATTCCGCGGGTCTGGAAGTTCTGT
TCCAGGGGCCCATGCAGGCGATCAAATGCGTAGTTGTCGGCGATGGTGCCGTCGGAA
AGACCTGTCTGCTGATTAGCTACACCACAAACGCGTTTCCTGGCGAATACATCCCAAC
CGTTTTCGACAACTATTCGGCGAATGTGATGGTTGATGGGAAACCCGTGAATCTGGGC
TTGTGGGATACTGCTGGTCAGGAGGATTACGATCGCTTACGCCCACTTAGCTATCCAC
AGACAGACGTCTTTCTGATCTGCTTTTCACTGGTGTCTCCCGCTTCCTTCGAAAACGTA
CGCGCCAAATGGTATCCGGAAGTTCGTCACCATTGCCCGAATACGCCGATTATCCTCG
TAGGTACCAAACTCGATTTGCGCGATGACAAAGACACGATTGAGAAACTGAAGGAAA
AGAAACTGACGCCGATTACCTATCCGCAAGGCTTAGCAATGGCCAAAGAGATTGGTG
CAGTCAAATATCTGGAATGCAGTGCACTGACTCAACGTGGGCTGAAAACCGTGTTCG
ATGAAGCGATTCGTGCGGTGTTATGTCCGCCTCCGGTGAAGAAACGGAAACGCAAAT
GTTTGCTGCTTTGA

KrasB

Protein sequence

MKHHHHHHHHHHSAGLEVLFQGPMTEYKLVVVGAGGVGKSALTIQLIQNHFVDEYDPT
IEDSYRKQVVIDGETCLLDILDTAGQEEYSAMRDQYMRTGEGFLCVFAINNTKSFEDIHH
YREQIKRVKDSEDVPMVLVGNKCDLPSRTVDTKQAQDLARSYGIPFIETSAKTRQGVDD
AFYTLVREIRKHKEKMSKDGKKKKKKSKTKCVIM*

DNA sequence

ATGAAACATCACCATCACCATCACCATCATCACCATTCCGCGGGTCTGGAAGTTCTGT
TCCAGGGGCCCATGACCGAATACAAACTGGTCGTAGTGGGTGCTGGTGGTGTTGGCA
AATCAGCGTTGACCATTCAGCTGATTCAGAATCACTTCGTGGATGAGTATGATCCGAC
AATTGAGGATAGCTATCGCAAACAGGTAGTGATTGACGGCGAAACTTGCCTCTTAGA
CATTCTGGATACCGCTGGGCAAGAAGAGTACTCTGCAATGCGCGATCAGTACATGCG
TACTGGTGAAGGCTTTCTGTGTGTCTTTGCGATCAACAATACCAAGAGCTTCGAAGAT
ATCCATCATTATCGGGAACAGATCAAACGTGTGAAAGACTCGGAAGATGTGCCAATG
GTCCTTGTTGGCAACAAATGCGATCTGCCTAGTCGTACCGTTGACACGAAACAAGCCC
AGGACTTAGCACGCAGTTATGGCATTCCGTTCATTGAAACATCCGCCAAAACCCGTCA
AGGAGTTGATGATGCGTTTTATACGCTGGTACGCGAAATCCGCAAACACAAAGAGAA
AATGTCGAAAGACGGGAAGAAGAAGAAAAAGAAAAGCAAAACGAAATGTGTGATCA
TGTGA

HRas

Protein sequence

MKHHHHHHHHHHSAGLEVLFQGPMTEYKLVVVGAGGVGKSALTIQLIQNHFVDEYDPT
IEDSYRKQVVIDGETCLLDILDTAGQEEYSAMRDQYMRTGEGFLCVFAINNTKSFEDIHQ
YREQIKRVKDSDDVPMVLVGNKCDLAARTVESRQAQDLARSYGIPYIETSAKTRQGVED
AFYTLVREIRQHKLRKLNPPDESGPGCMSCKCVLS*

DNA sequence

ATGAAACATCACCATCACCATCACCATCATCACCATTCCGCGGGTCTGGAAGTTCTGT
TCCAGGGGCCCATGACCGAATACAAGCTGGTGGTCGTAGGGGCTGGAGGTGTTGGCA
AAAGTGCGTTGACCATCCAGCTGATTCAGAACCACTTCGTGGATGAGTACGACCCCA
CCATTGAAGATAGCTATCGCAAACAGGTCGTGATTGACGGAGAGACTTGTTTGCTGG
ACATTCTGGATACAGCTGGCCAAGAAGAGTATAGCGCAATGCGCGATCAGTACATGC
GTACCGGTGAAGGCTTTCTGTGCGTGTTTGCGATCAACAACACGAAATCCTTTGAGGA
CATCCATCAGTATCGCGAACAGATCAAACGCGTCAAAGACAGCGATGATGTGCCGAT
GGTTCTGGTTGGGAATAAGTGCGATTTAGCCGCACGTACGGTTGAATCGCGTCAAGC
GCAAGATCTTGCCCGTTCCTATGGCATTCCGTATATCGAAACCTCTGCCAAAACGCGT
CAAGGTGTAGAAGATGCGTTCTACACTCTGGTACGCGAAATTCGGCAGCATAAACTG
CGCAAACTCAATCCGCCAGACGAAAGTGGTCCTGGCTGTATGTCGTGTAAGTGCGTG
TTATCATGA

Cdc42

Protein sequence

MKHHHHHHHHHHSAGLEVLFQGPMQTIKCVVVGDGAVGKTCLLISYTTNKFPSEYVPTV
FDNYAVTVMIGGEPYTLGLFDTAGQEDYDRLRPLSYPQTDVFLVCFSVVSPSSFENVKEK

WVPEITHHCPKTPFLLVGTQIDLRDDPSTIEKLAKNKQKPITPETAEKLARDLKAVKYVEC
SALTQKGLKNVFDEAILAALEPPEPKKSRRCVLL*

DNA sequence

ATGAAACATCACCATCACCATCACCATCATCACCATTCCGCGGGTCTGGAAGTTCTGT
TCCAGGGGCCCATGCAGACCATCAAATGCGTAGTCGTGGGTGATGGAGCAGTTGGCA
AAACGTGTCTGCTCATCTCCTATACCACCAACAAATTTCCCAGCGAATACGTGCCTAC
GGTATTCGACAATTACGCGGTTACCGTCATGATTGGTGGTGAACCCTATACCTTGGGC
CTGTTCGATACTGCAGGCCAAGAGGACTATGATCGCTTACGCCCTCTGTCGTATCCGC
AAACCGACGTCTTTCTTGTGTGCTTTAGCGTTGTGTCTCCGAGTTCGTTCGAAAACGT
GAAAGAGAAATGGGTACCGGAAATTACGCACCATTGTCCGAAAACTCCGTTTCTGCT
GGTTGGCACACAGATCGATCTGCGCGATGATCCAAGCACCATTGAGAAACTTGCCAA
GAACAAACAGAAACCGATTACGCCAGAAACTGCGGAGAAATTAGCCCGTGATCTGA
AAGCCGTCAAGTACGTGGAATGCTCAGCTTTGACACAGAAAGGGCTGAAGAATGTGT
TTGACGAAGCGATTCTGGCTGCGTTAGAACCGCCAGAACCGAAGAAAAGTCGTCGGT
GTGTTCTCCTGTGA

mCherry-Cdc42

Protein sequence

MKHHHHHHHHHHSAGLEVLFQGPMVSKGEEDNMAIIKEFMRFKVHMEGSVNGHEFEIE
GEGEGRPYEGTQTAKLKVTKGGPLPFAWDILSPQFMYGSKAYVKHPADIPDYLKLSFPEG
FKWERVMNFEDGGVVTVTQDSSLQDGEFIYKVKLRGTNFPSDGPVMQKKTMGWEASSE
RMYPEDGALKGEIKQRLKLKDGGHYDAEVKTTYKAKKPVQLPGAYNVNIKLDITSHNED
YTIVEQYERAEGRHSTGGMDELYKEFMQTIKCVVVGDGAVGKTCLLISYTTNKFPSEYVP
TVFDNYAVTVMIGGEPYTLGLFDTAGQEDYDRLRPLSYPQTDVFLVCFSVVSPSSFENVK
EKWVPEITHHCPKTPFLLVGTQIDLRDDPSTIEKLAKNKQKPITPETAEKLARDLKAVKYV
ECSALTQKGLKNVFDEAILAALEPPEPKKSRRCVLL*

DNA sequence

ATGAAACATCACCATCACCATCACCATCATCACCATTCCGCGGGTCTGGAAGTTCTGT
TCCAGGGGCCCATGGTGAGCAAGGGCGAGGAGGATAACATGGCCATCATCAAGGAG
TTCATGCGCTTCAAGGTGCACATGGAGGGCTCCGTGAACGGCCACGAGTTCGAGATC
GAGGGCGAGGGCGAGGGCCGCCCCTACGAGGGCACCCAGACCGCCAAGCTGAAGGT
GACCAAGGGTGGCCCCCTGCCCTTCGCCTGGGACATCCTGTCCCCTCAGTTCATGTAC
GGCTCCAAGGCCTACGTGAAGCACCCCGCCGACATCCCCGACTACTTGAAGCTGTCCT
TCCCCGAGGGCTTCAAGTGGGAGCGCGTGATGAACTTCGAGGACGGCGGCGTGGTGA
CCGTGACCCAGGACTCCTCCCTGCAGGACGGCGAGTTCATCTACAAGGTGAAGCTGC
GCGGCACCAACTTCCCCTCCGACGGCCCCGTAATGCAGAAGAAGACCATGGGCTGGG
AGGCCTCCTCCGAGCGGATGTACCCCGAGGACGGCGCCCTGAAGGGCGAGATCAAGC
AGAGGCTGAAGCTGAAGGACGGCGGCCACTACGACGCTGAGGTCAAGACCACCTAC
AAGGCCAAGAAGCCCGTGCAGCTGCCCGGCGCCTACAACGTCAACATCAAGTTGGAC
ATCACCTCCCACAACGAGGACTACACCATCGTGGAACAGTACGAACGCGCCGAGGGC
CGCCACTCCACCGGCGGCATGGACGAGCTGTACAAGGAATTTATGCAGACCATCAAA
TGCGTAGTCGTGGGTGATGGAGCAGTTGGCAAAACGTGTCTGCTCATCTCCTATACCA
CCAACAAATTTCCCAGCGAATACGTGCCTACGGTATTCGACAATTACGCGGTTACCGT
CATGATTGGTGGTGAACCCTATACCTTGGGCCTGTTCGATACTGCAGGCCAAGAGGA

CTATGATCGCTTACGCCCTCTGTCGTATCCGCAAACCGACGTCTTTCTTGTGTGCTTTA
GCGTTGTGTCTCCGAGTTCGTTCGAAAACGTGAAAGAGAAATGGGTACCGGAAATTA
CGCACCATTGTCCGAAAACTCCGTTTCTGCTGGTTGGCACACAGATCGATCTGCGCGA
TGATCCAAGCACCATTGAGAAACTTGCCAAGAACAAACAGAAACCGATTACGCCAGA
AACTGCGGAGAAATTAGCCCGTGATCTGAAAGCCGTCAAGTACGTGGAATGCTCAGC
TTTGACACAGAAAGGGCTGAAGAATGTGTTTGACGAAGCGATTCTGGCTGCGTTAGA
ACCGCCAGAACCGAAGAAAAGTCGTCGGTGTGTTCTCCTGTAA

MSP1E3D1

Protein sequence

MGSSHHHHHHENLYFQGSTFSKLREQLGPVTQEFWDNLEKETEGLRQEMSKDLEEVKA
KVQPYLDDFQKKWQEEMELYRQKVEPLRAELQEGARQKLHELQEKLSPLGEEMRDRAR
AHVDALRTHLAPYLDDFQKKWQEEMELYRQKVEPLRAELQEGARQKLHELQEKLSPLG
EEMRDRARAHVDALRTHLAPYSDELRQRLAARLEALKENGGARLAEYHAKATEHLSTL
SEKAKPALEDLRQGLLPVLESFKVSFLSALEEYTKKLNTQ*

DNA sequence

ATGGGCAGCAGCCATCATCATCATCATCATGAAAACCTGTATTTTCAGGGCAGCACCT
TTAGCAAACTGCGTGAACAGCTGGGCCCGGTGACCCAGGAATTTTGGGATAACCTGG
AAAAAGAAACCGAAGGCCTGCGTCAGGAAATGAGCAAAGATCTGGAAGAGGTGAAA
GCGAAAGTGCAGCCGTATCTGGATGACTTTCAGAAAAAATGGCAGGAAGAGATGGA
ACTGTATCGTCAGAAAGTGGAACCGCTGCGTGCGGAACTGCAGGAAGGCGCGCGTCA
GAAACTGCATGAACTGCAGGAAAAACTGAGCCCGCTGGGCGAAGAGATGCGTGATC
GTGCGCGTGCGCATGTGGATGCGCTGCGTACCCATCTGGCGCCGTATCTGGATGACTT
TCAGAAAAAATGGCAGGAAGAGATGGAACTGTATCGTCAGAAAGTGGAACCGCTGC
GTGCGGAACTGCAGGAAGGCGCGCGTCAGAAACTGCATGAACTGCAGGAAAAACTG
AGCCCGCTGGGCGAAGAGATGCGTGATCGTGCGCGTGCGCATGTGGATGCGCTGCGT
ACCCATCTGGCGCCGTATAGCGATGAACTGCGTCAGCGTCTGGCGGCCCGTCTGGAA
GCGCTGAAAGAAAACGGCGGTGCGCGTCTGGCGGAATATCATGCGAAAGCGACCGA
ACATCTGAGCACCCTGAGCGAAAAAGCGAAACCGGCGCTGGAAGATCTGCGTCAGG
GCCTGCTGCCGGTGCTGGAAAGCTTTAAAGTGAGCTTTCTGAGCGCGCTGGAAGAGT
ATACCAAAAAACTGAACACCCAGTAA

Fiji Macro Code

Z-stack analysis macro code:

```
setPasteMode("Copy");
originalImage = getTitle();

getDimensions(width, height, channels, slices, frames);
newImage(originalImage + "_membraneSliceTimeSeries", bitDepth+"-bit", width, height, channels,
1, frames);
membraneSliceTimeSeries = getTitle();

for(t=1; t<=frames; t++) {
	selectWindow(originalImage);
	//Select the first channel, slice and frame t.
	Stack.setPosition(1,1,t);
	brightestMean = 0;
	brightestSlice = 1;
	for(i=1; i<slices; i++) {
		Stack.setPosition(1,i,t);
		getStatistics(area, mean);
		if(brightestMean<mean) {
			brightestMean = mean;
			brightestSlice = i;
		}
	}
	//print(brightestSlice); //test
	Stack.setPosition(1,brightestSlice,t);
	run("Select All");
	run("Copy");
	selectWindow(membraneSliceTimeSeries);
	Stack.setPosition(1,1,t);
	run("Paste");
	//print the second channel
	selectWindow(originalImage);
	Stack.setPosition(2,brightestSlice,t);
	run("Select All");
	run("Copy");
	selectWindow(membraneSliceTimeSeries);
	Stack.setPosition(2,1,t);
	run("Paste");
}
```

Supplementary References

(1) Dursina, B.; Reents, R.; Delon, C.; Wu, Y. W.; Kulharia, M.; Thutewohl, M.; Veligodsky, A.; Kalinin, A.; Evstifeev, V.; Ciobanu, D.; et al. Identification and specificity profiling of protein prenyltransferase inhibitors using new fluorescent phosphoisoprenoids. J. Am. Chem. Soc. 2006, 128 (9), 2822-2835. DOI: 10.1021/ja052196e.
(2) Li, Y.; Wang, E. D.; Wang, Y. L. A modified procedure for fast purification of T7 RNA polymerase. Protein Expres. Purif. 1999, 16 (2), 355-358. DOI: 10.1006/prep.1999.1083.
